# The neuroimmune nexus: unraveling the role of the mtDNA-cGAS-STING signal pathway in Alzheimer’s disease

**DOI:** 10.1186/s13024-025-00815-2

**Published:** 2025-03-04

**Authors:** Shuiyue Quan, Xiaofeng Fu, Huimin Cai, Ziye Ren, Yinghao Xu, Longfei Jia

**Affiliations:** Innovation Center for Neurological Disorders and Department of Neurology, Xuanwu Hospital, Capital Medical University, National Clinical Research Center for Geriatric Diseases, 45 Changchun St, Beijing, 100053 China

**Keywords:** MtDNA, cGAS, STING, Neuroinflammation, Alzheimer’s disease, Treatment

## Abstract

The relationship between Alzheimer's disease (AD) and neuroimmunity has gradually begun to be unveiled. Emerging evidence indicates that cyclic GMP-AMP synthase (cGAS) acts as a cytosolic DNA sensor, recognizing cytosolic damage-associated molecular patterns (DAMPs), and inducing the innate immune response by activating stimulator of interferon genes (STING). Dysregulation of this pathway culminates in AD-related neuroinflammation and neurodegeneration. A substantial body of evidence indicates that mitochondria are involved in the critical pathogenic mechanisms of AD, whose damage leads to the release of mitochondrial DNA (mtDNA) into the extramitochondrial space. This leaked mtDNA serves as a DAMP, activating various pattern recognition receptors and immune defense networks in the brain, including the cGAS-STING pathway, ultimately leading to an imbalance in immune homeostasis. Therefore, modulation of the mtDNA-cGAS-STING pathway to restore neuroimmune homeostasis may offer promising prospects for improving AD treatment outcomes. In this review, we focus on the mechanisms of mtDNA release during stress and the activation of the cGAS-STING pathway. Additionally, we delve into the research progress on this pathway in AD, and further discuss the primary directions and potential hurdles in developing targeted therapeutic drugs, to gain a deeper understanding of the pathogenesis of AD and provide new approaches for its therapy.

## Background

Alzheimer’s disease (AD) is the most common form of chronic neurodegenerative disease, posing significant health risks to the elderly [1]. Clinically, it primarily manifests as a progressive impairment of memory and cognitive function [2]. Analysis of epidemiological data has indicated that the global prevalence of dementia is expected to triple by 2050, imposing heavy economic and psychological challenges on communities and households [3–5]. The primary pathological features of AD involve the widespread amyloid plaques and neurofibrillary tangles (NFT) within the brain, triggering irreversible neuronal damage and cognitive decline [6]. Despite significant global financial investments for tackling AD, effective treatment strategies are still lacking [7]. One possible reason for this is that the etiology of AD is multifactorial [8], and the precise molecular mechanisms underlying its onset and progression remain largely elusive. As such, there is an urgent need to explore new disease-modifying therapies that target the critical processes of AD pathogenesis [9], with the aim of obtaining better treatment outcomes. Recent studies have identified the involvement of maladaptive inflammatory responses and immune dysregulation in AD development [10, 11], highlighting the importance of understanding the potential molecular mechanisms underlying immune-inflammatory reactions.

The innate immune, serving as the first line of defense in the body’s immune system, can distinguish between “self” and “non-self” effectively [12]. Endogenous damage-associated molecular patterns (DAMPs) released after cellular damage, such as self-proteins or nucleic acids, can also be recognized by the innate immune system, thereby eliciting a swift and non-specific immune response [13]. This recognition process is mediated by pattern recognition receptors (PRRs), the majority of which possess sequence- and cell-type- specificity [14]. Notably, as a unique type of PRRs, cyclic GMP-AMP synthase (cGAS) lacks DNA sequence specificity, and cannot effectively distinguish between self and foreign DNA [15]. Consequently, mitochondrial DNA (mtDNA) and other double-stranded DNA (dsDNA) can act as DAMPs to bind cGAS upon entry into the cytoplasm, thus activating the downstream stimulator of interferon genes (STING) [16]. Typically, the cGAS-STING pathway serves as a protective mechanism against invading pathogens, and its activation by external nucleic acids, such as those from viruses, can effectively protect the cells from potential threats [17]. However, overactivation of this pathway, triggered by the accumulation of ectopic dsDNA in the central nervous system (CNS), may exacerbate neuroinflammatory responses. This dysregulated inflammatory environment likely further impairs neuronal cells, causing damage to healthy tissues and potentially triggering multiple neurological diseases that have been reported to be associated with abnormal inflammation, including AD, Parkinson’s disease (PD) [18], amyotrophic lateral sclerosis (ALS) [19], postoperative cognitive dysfunction (POCD) [20], and traumatic brain encephalopathy (TBE) [21], although the pathogenesis of these diseases shows high heterogeneity. Recent studies particularly highlight its critical role in AD.

In this review, we summarize how mtDNA is released into extramitochondrial space under cellular stress, and subsequently recognized by the cGAS-STING signal pathway. Furthermore, we discuss the pivotal role of the mtDNA-cGAS-STING pathway in AD and the prospects of targeting this pathway for AD treatment. We believe that an in-depth exploration of the mechanisms related to this cascade pathway will provide greater hope for delaying AD progression.

## Mechanisms of mtDNA release

In mammalian cells, mtDNA is a double-stranded, circular DNA comprising 16,569 base pairs and encoding 37 genes [22]. mtDNA is distributed throughout the mitochondrial network, and the proteins it encodes are involved in a wide range of physiological functions [23], including energy supply, Ca^2+^ homeostasis maintenance, synaptic plasticity [24], and heme and cholesterol synthesis [25]. Notably, mtDNA itself exerts an endogenous pro-inflammatory effect, which is largely attributed to its unmethylated CpG islands, akin to bacterial genomes [26, 27]. As such, mtDNA released outside of mitochondria can act as an immune stimulant to mediate immune-inflammatory processes. Nevertheless, mtDNA possesses an intricate set of self-protection systems that protect it from recognition by the immune surveillance system under physiological conditions, thus maintaining its relatively stable existence within cells. These mechanisms encompass the barrier of the mitochondrial “double-layer” membrane, balanced mitochondrial dynamics, coordinated regulation of various nuclear genes, mitochondrial autophagy, and interplay with other cellular organelles [28–30]. Abnormalities in these mechanisms may lead to mtDNA mutations or relocation outside the mitochondria, although the specific release process and related regulatory mechanisms remain unclear. Interestingly, the mechanisms underlying mtDNA releasing to extramitochondrial space differ between apoptotic and living cells; in cells under apoptotic stress, mtDNA escape predominantly occurs through macropores formed by apoptotic regulators (BAK/BAX) [31, 32], including in senescent cells that remain resistant to apoptosis, where mtDNA can also be released via BAK/BAX-dependent minority MOMP (miMOMP) [33]. Whereas in living cells, mtDNA primarily depends on voltage-dependent anion channel (VDAC) oligomerization pores in the outer mitochondrial membrane (OMM) that promote mitochondrial outer membrane permeabilization (MOMP) [34, 35]. Besides, gasdermin pores, mitochondrial dynamics, extracellular vesicles (EVs), and neutrophil extracellular traps (NETs) also contribute to mtDNA release [36]. As such, mtDNA escape is an exceedingly complex process involving an interplay of multiple mechanisms. In the following sections, we systematically summarize these mechanisms of mtDNA release to better understand the role of mtDNA in inflammatory diseases and immune regulation.

### The mechanism of mtDNA release into the extramitochondrial space

#### BAK/BAX pores

The prevailing view supports the notion that MOMP is the primary trigger of mtDNA release. When apoptotic signals occur within cells, the pro-apoptotic factors BAX and BAK are activated and recruited to oligomerize on the OMM, forming macropores that induce MOMP [37]. The growth rate of these macropores and the relative kinetics of mtDNA are influenced by the relative availability of Bax and Bak, while their interactions can regulate the dynamics of mtDNA release [38, 39]. Unlike non-inflammatory mitochondrial apoptosis, which relies on caspase activation, mtDNA release through these macropores does not require activated caspases [32]. The absence of the latter is crucial for mtDNA-mediated inflammation. If caspases are activated, cell death occurs after MOMP, without triggering the type I interferon (IFN) response [40, 41]. In other words, caspase activation can sequester the apoptotic process of cells from the immune system, thus avoiding unnecessary inflammatory responses. This precise regulatory mechanism ensures the orderly progression of apoptosis, which is important for maintaining immune homeostasis in organisms.

Following MOMP, the inner mitochondrial membrane (IMM) protrudes into the cytoplasm through BAK/BAX macropores to form mitochondrial herniations. Then, the mitochondrial matrix contents, including the mitochondrial genome and other mitochondrial components, are translocated outside the mitochondria [31], subsequently activating caspase-independent cell death (CICD)-associated inflammation. This process may further result in a loss of IMM integrity, culminating in mitochondrial inner membrane permeabilization (MIMP) [32]. Additionally, studies have indicated that mtDNA is released into the cytoplasm in the form of entire nucleoids. However, whether these released nucleoids remain intact or fragmented remains unclear [42]. Clarifying the fate and effects of these released nucleoids within the cytoplasm is crucial, as they may be closely linked to the potential of mtDNA as an effective biomarker.

Notably, MOMP may also be associated with the release of mitochondrial double-stranded RNA (mt-dsRNA) [43]. Dhir et al. found that the release of mt-dsRNA is almost entirely blocked by the downregulation of BAX and BAK, indicating that mt-dsRNA release may predominantly occur via MOMP. These released mt-dsRNAs can also be recognized by dsRNA sensors and activate the IFN-I pathway [44]. Furthermore, they may act as mitochondrial self-antigens involved in the development of autoimmune diseases [45]. Nevertheless, the mechanisms underlying mt-dsRNA release remain poorly understood, and their precise role in immune responses requires further investigation.

#### VDAC oligomer pores

In living cells, the mechanism by which mtDNA is released is not uniform. Multiple studies have indicated that oxidative stress, when insufficient to activate BAK and BAX, can induce VDAC oligomerization and subsequently form pores in the OMM, thus promoting MOMP in living cells and facilitating mtDNA release [34]. Notably, mtDNA translocation through VDAC requires the coordination of other proteins, particularly the adenine nucleotide translocator (ANT) located in the IMM, and Cyprin D in the matrix. These proteins are considered key components of the mitochondrial permeability transition pore (mPTP), a diverse protein complex located between the IMM and OMM [46, 47]. García et al. provided evidence to support this view, showing that cyclosporin A, a Cyprin D inhibitor, could affect the permeability transition pore and inhibit mtDNA release into the cytoplasm by 52% [48]. Unlike the BAK/BAX pores, mtDNA fragments, which are components of specific genes, are released via VDAC pores [49]. In addition, mPTP pores are expected to allow only mtDNA fragments with a molecular weight of less than 1.5 kDa to pass through [50], enriching evidence that the mtDNA released via VDAC pores are fragments rather than intact nucleoids.

The formation of VDAC oligomer pores is a complex process regulated by several factors. Upon entry into the mitochondrial intermembrane space, mtDNA fragments themselves interact with residues at the N-terminus of the VDAC1 subtype, thus promoting VDAC oligomerization and forming a feed-forward loop [34]. The virus-related kinase 2 (VRK2) protein, a member of the vaccinia virus-related kinase family and a serine/threonine kinase with catalytic activity [51, 52], is considered another important regulator of VDAC1 oligomerization during mtDNA release. VRK2 was recently shown to induce cGAS-mediated innate immune response [53], indicating its potential as a therapeutic target for infectious and autoimmune diseases associated with mtDNA release. Additionally, Baik et al. demonstrated that the dissociation of hexokinase 2 (HK2) from VDAC triggers calcium release from the endoplasmic reticulum (ER), which is subsequently taken up by the mitochondria, leading to VDAC oligomerization and mtDNA escape [54]. However, these studies only partly elucidated the regulatory mechanisms governing mtDNA release through VDAC oligomeric pores, and their findings are likely limited to specific disease contexts. Further research is therefore needed to investigate whether there are differences in the regulation of mtDNA release through VDAC oligomeric pores under various disease conditions such as infection, autoimmune diseases, and cancer, which will help to identify specific therapeutic targets to provide more possibilities for the treatment of these related diseases.

#### Gasdermin pores

Gasdermins (GSDMs) are a class of pore-forming proteins that represent another channel for mtDNA escape from mitochondria [55]. As a pivotal effector molecule in pyroptosis, gasdermin undergoes cleavage by activated caspases to generate an N-terminal domain, that inserts into the plasma membrane and oligomerizes to form large pores through its pore-forming activity [56], allowing the release of inflammatory substances like interleukin-1β (IL-1β) [57]. Huang et al. indicated that mtDNA can be released into the cytosol through gasdermin pores to activate downstream signaling pathways during inflammatory injury [58]. In detail, several members of the gasdermins, including gasdermin A3, gasdermin D (GSDMD), and gasdermin E, can facilitate mtDNA release during apoptosis or pyroptosis, and inhibit the pore activity of GSDMD significantly impedes this process, consequently ameliorating mitochondrial network structure damage and rescuing mitochondrial dysfunction [55, 59]. Additionally, oxidized mtDNA can directly interact with the GSDMD-N domain to enhance its oligomerization during the pore-forming process [60], which may exacerbate mitochondrial membrane rupture. Furthermore, membrane damage induced by gasdermin could precipitate the collapse of the mitochondrial network system, potentially providing alternative pathways for mtDNA release, such as NETs [61], and ultimately resulting in the displacement of mtDNA outside the mitochondria or even extracellularly. These studies indicate a plausible interplay between gasdermin and damaged mitochondria, both promoting cell death. Overall, the formation of gasdermin pores is a significant determinant of mtDNA escape, extending its importance beyond pyroptosis.

### The mechanism and significance of mtDNA release into the extracellular space

Diverse mtDNA forms have been detected in different biological fluids, such as plasma [62], serum [63], cerebrospinal fluid (CSF) [64], and synovial fluid [65], indicating the further release of mtDNA from the cytoplasmic compartment into the extracellular environment. Currently, the active release of mtDNA is believed to occur primarily through transportation via extracellular EVs or as components of NETs, whereas its passive release typically occurs during cell injury and death processes like apoptosis, necrosis, and pyroptosis [25]. Recent studies have suggested that the level of cell-free mtDNA (cf-mtDNA) released into the extracellular fluid is a significant biomarker that partly reflects the severity and prognosis of various inflammation-related diseases. For example, a correlation has been observed between higher mtDNA copy numbers (mtDNAcn) in circulation and better cognitive function [66]. Similarly, patients with coronary heart disease have lower mtDNA levels than the control group [67]. Interestingly, evidence suggests that only cf-mtDNA, and not mtDNA present in intact circulating mitochondria, can elicit an inflammatory response [68]. Understanding the mechanisms underlying mtDNA releasing into extracellular space and the pathophysiological functions of its various forms is essential to advance our knowledge of mitochondrial biology and its roles in health and disease.

## Activation of cGAS-STING by Leaked mtDNA

**Fig. 1:**
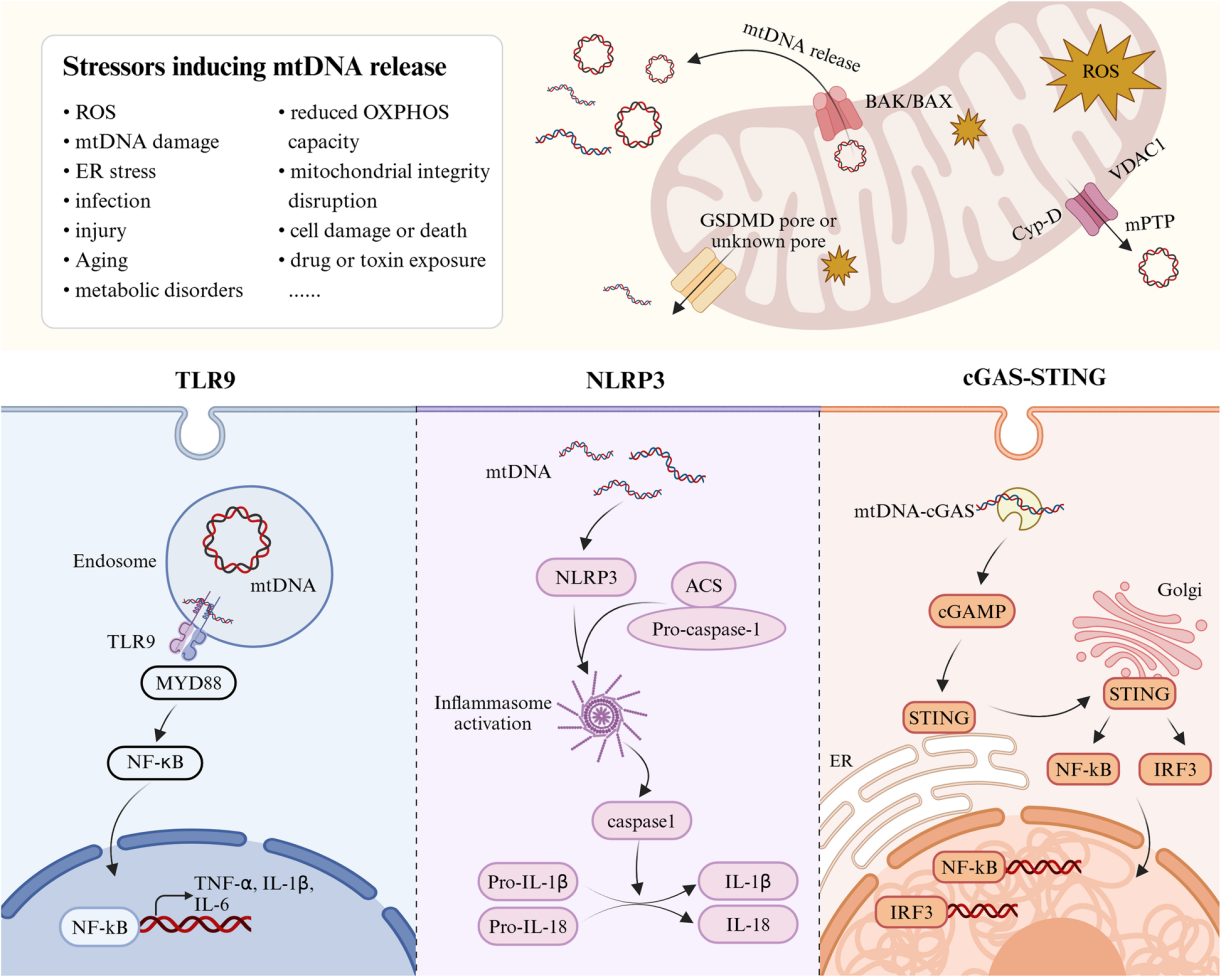
Overview of the mechanisms of mtDNA release and its effect on the innate immune response. Under normal circumstances, mtDNA is encapsulated within mitochondria without disturbing the immune system. However, when exposed to various adverse factors such as ROS, cellular stress, and aging, mitochondria inevitably incur a certain degree of damage. At this point, mtDNA is released into the extramitochondrial space through mechanisms involving BAK/BAX, VDAC1, GSDMD, and others. Subsequently, the leaked mtDNA acts as DAMPs, recognized by diverse PRRs, primarily activating the body’s innate immune response through three different inflammatory pathways: TLR9, NLPR3, and cGAS-STING

As mentioned above, under physiological circumstances, mtDNA is encapsulated within the “double membrane” structure of mitochondria and does not disturb the immune system. Upon release into the cytoplasm or circulation, it acts as a potential immune stimulus, eliciting an inherent immune response in the body. Although the precise mechanisms by which mtDNA triggers inflammation have not yet been fully elucidated, it is widely recognized that mtDNA primarily affects immune responses through three important signaling pathways: the toll-like receptor-9 (TLR-9), cGAS-STING, and nucleotide-binding oligomerization domain (NOD), leucine-rich repeat (LRR), and pyrin domain-containing protein 3 (NLRP3) inflammasome pathways [69] (Fig. 1). Given that the association of TLR-9 and NLRP3 with mtDNA has been extensively summarized in many other excellent reviews [25, 70], herein, we primarily focused on the crosstalk between mtDNA and the emerging immune pathway cGAS-STING, which is increasingly being recognized for its role in AD.

### Molecular mechanisms of the cGAS-STING pathway

**Fig. 2:**
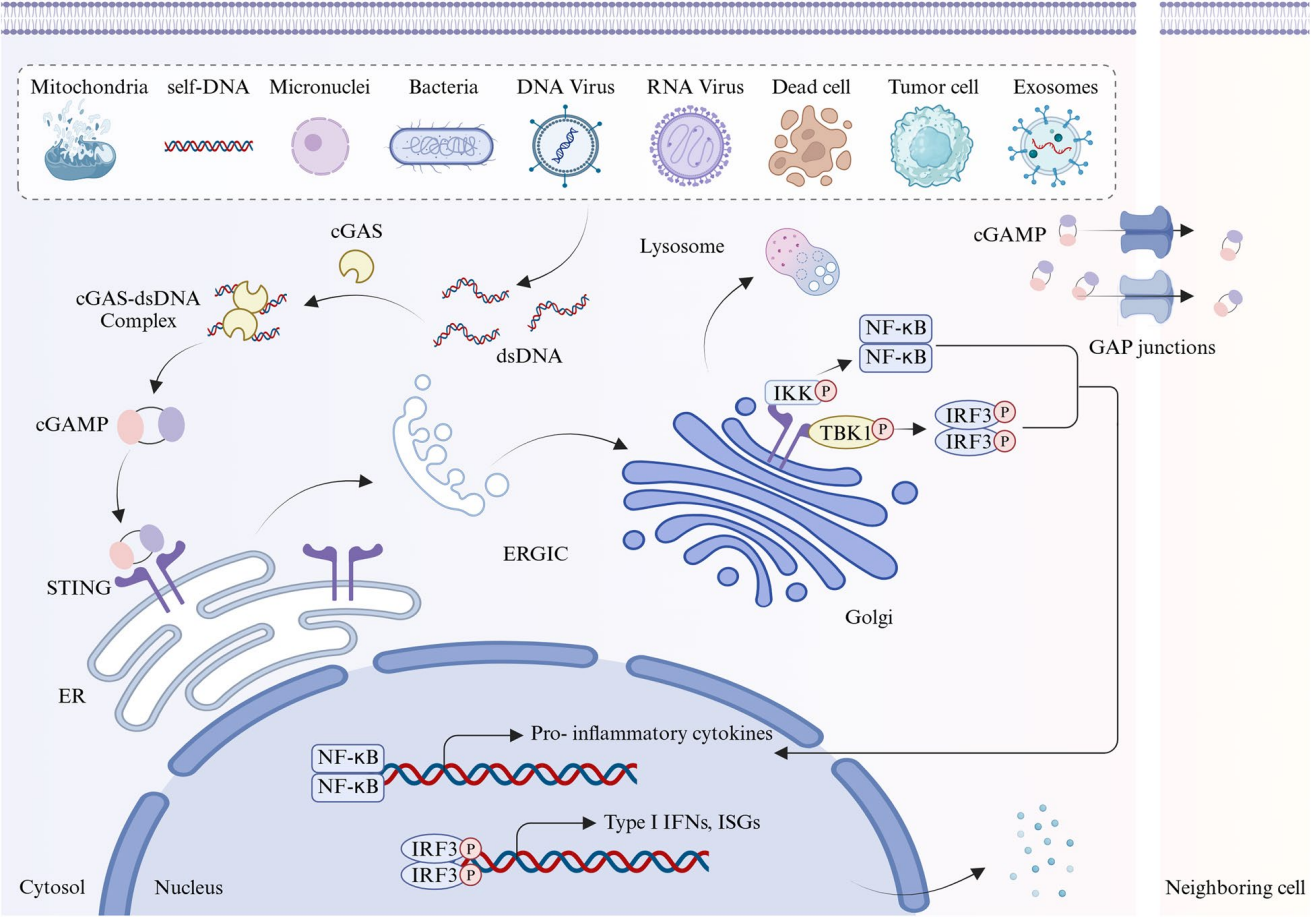
Overview of the molecular mechanisms of the cGAS-STING signal pathway. The activation of the cGAS-STING signaling begins with the detection of abnormal dsDNA within the cell. Initially, dsDNA binds to cGAS, triggering its enzymatic activity and the production of 2’3’-cGAMP. Subsequently, cGAMP binds to STING on the ER membrane, inducing conformational changes that result in STING oligomerization and translocation to the ERGIC and Golgi apparatus. This change leads to the activation of signaling molecules such as TBK1 and IRF3, which enter the nucleus and initiate the transcription of immune factors like interferons, ultimately initiating the body’s immune response to combat infection and damage

Activation of the cGAS-STING pathway is an extremely complex process (Fig. 2). The molecular weight of cGAS is approximately 60 kDa, and it comprises an N-terminal region that mediates binding to cell membrane, a highly conserved nucleotidyltransferase core domain, and a male abnormal gene family 21 (Mab21) nucleotidyltransferase domain [71, 72]. The Mab21 domain is primarily involved in dsDNA binding, and structural defects in it cause cGAS to lose the ability to induce IFN-β expression [73]. Thus, maintaining structural integrity is especially important for the physical function of cGAS. Unlike other PRRs, cGAS binds to negatively charged dsDNA through electrostatic interactions and hydrogen bonds, which partly explains its lack of sequence-specific recognition [74, 75]. In other words, it can bind to various types of nucleic acids, such as dsDNA, RNA:DNA hybrids, ssDNA, and dsRNA, but the latter two lack the ability to rearrange the catalytic pocket of cGAS to activate it [76, 77]. In addition, the intracellular localization of cGAS remains controversial. Although most studies consider it a “cytoplasmic sensor” [78], recent research indicated that cGAS is present in the cell nucleus and membrane as well [79–82]. For example, the localization of cGAS is cell-cycle dependent [83, 84]; it is typically located in the cytoplasm of non-dividing cells but translocates into the nucleus during mitosis in proliferating cells, where it binds to chromatin DNA [85]. In summary, its subcellular localization is tightly related to the cell types and biological contexts, allowing it to function more effectively.

Indeed, cGAS possesses catalytic inertness. When it binds to dsDNA, a conformational change occurs, converting the catalytic pocket of cGAS from an inactive “closed” conformation to an active “open” conformation, forming a 2:2 dimeric structure composed of cGAS and dsDNA [75, 86]. Subsequently, this complex catalyzes the formation of 2’3’-cyclic GMP-AMP (2’3’-cGAMP) from guanosine triphosphate (GTP) and adenosine triphosphate (ATP), which serves as a second messenger, binding and activating the adaptor protein STING located on the ER membrane, initiating a cascade of intracellular signaling events crucial for the immune response [87].

Ishikawa et al. first reported the importance of STING in the innate immune response to antiviral immunity. As an important component of nonspecific immunity against aberrant cytoplasmic DNA, STING is widely expressed in the ER across various cell types [88]. Upon binding to 2’3’-cGAMP, STING oligomerizes and is trafficked from the ER to the Golgi apparatus [89, 90]. During this process, the C-terminal tail (CCT) of STING recruits and activates TANK-binding kinase 1 (TBK1), which phosphorylates STING and interferon regulatory factor 3 (IRF3) [91]. Subsequently, phosphorylated IRF3 forms dimers and is translocated to the nucleus to bind IFN response elements, inducing extensive transcription of downstream type I interferons (IFN-I) and interferon-stimulated genes (ISGs), thus triggering IFN-I-mediated immune responses [92]. Additionally, STING can also phosphorylate IκB kinase (IKK), causing the release of nuclear factor kappa-B (NF-κB) into the nucleus, activating the classical NF-κB signaling pathway, and inducing the expression of genes such as tumor necrosis factor-alpha (TNF-α), interleukin-6 (IL-6), and IL-1β, triggering a broad immune response [93].

cGAS-STING-mediated autophagy represents the primitive and highly conserved function of this pathway [94]. Upon binding with cGAMP, STING buds from the ER to form COPII vesicles, forming the ER-Golgi intermediate compartment (ERGIC). The ERGIC-containing STING serves as a membrane source for microtubule-associated protein 1 light chain 3 (LC3) lipidation, promoting the formation of autophagosomes [95, 96]. In contrast, autophagy can feedback-regulate the activity of this pathway as well [97]. In addition to negatively regulating STING signaling by interfering with the assembly of STING-TBK1-IRF3 or STING degradation, autophagy prevents STING activation by delivering cytoplasmic DNA to lysosomal degradation [95, 98, 99]. The reciprocal regulation between the cGAS-STING pathway and autophagy helps clear damaged organelles, abnormal proteins, and accumulated dsDNA in the cytoplasm, suppressing unnecessary inflammatory responses and supporting cellular homeostasis.

### Crosstalk between cGAS-STING and mtDNA

The interaction between cGAS-STING and mtDNA and its involvement in disease pathogenesis has long been a focal point of research. A pivotal discovery linking mtDNA to the cGAS-STING pathway demonstrates that mtDNA stress is an intrinsic trigger of antiviral responses. Specifically speaking, viral infection can lead to abnormal mtDNA escape into the cytosol, where it binds to cGAS and promotes STING-IRF3-dependent signaling, mediating antiviral innate immunity [100]. Other studies have also provided evidence of mtDNA colocalization with cGAS and triggering inflammatory damage under specific conditions [40, 101]. Sliter et al. presented compelling findings connecting mtDNA-STING signaling to neurodegenerative diseases, showing that mitochondrial stress induced by mtDNA mutations triggers a STING-IFN-I response in a PD mouse model lacking Parkin or PINK1 [102]. Furthermore, the release of mtDNA is related to the mitochondrial localization of neuropathological proteins, which may impair the integrity and function of the mitochondrial membrane. A critical study has reported that the mitochondrial localization of tau protein triggers mtDNA leakage and cGAS activation in microglia treated with tau fibrils, diminishing cognitive resilience through decreasing the neuronal transcriptional network of myocyte enhancer factor 2c (MEF2C) [103]. A similar mechanism was revealed in a parallel study, where the mitochondrial translocation of transactive response DNA binding protein of 43 kDa (TDP-43) drives mtDNA release into the cytoplasm, activating the cGAS-STING pathway in ALS models [19]. These studies together demonstrate the interaction of mtDNA with the cGAS-STING and its downstream pathways, providing persuasive evidence that mtDNA escaping into the extramitochondrial space can be sensed by cGAS.

Further in-depth research has established the link between mitochondrial stress in neurons and neuroinflammation. After ischemia–reperfusion injury, ox-mtDNA activated the cGAS-STING pathway within neurons, and restricting the release of ox-mtDNA into the cytoplasm downregulated p-STING expression levels in both neurons and microglia [104]. Other research has reported that neurons could release vesicles containing mtDNA into the extracellular space, which can be engulfed by neighboring microglial cells [105–108]. Additionally, transfection of extracted mtDNA into microglia upregulated the expression of IFN-β which was significantly inhibited upon cGAS knockout [109], thus cGAS in microglia may also be activated by mtDNA released from other cells. Based on these results, we cautiously propose that stressed neurons can release mtDNA and other pro-inflammatory factors into the extracellular space under various harmful conditions, and this displaced mtDNA may then be recognized by nearby microglia or astrocytes, consequently activating cGAS-STING signaling in these immune cells and exacerbating neuroinflammation. Unfortunately, direct evidence for this process is still lacking in AD. If further validated, targeting this pathway may hold even greater promise.

## The role of the mtDNA-cGAS-STING pathway in AD

**Fig. 3:**
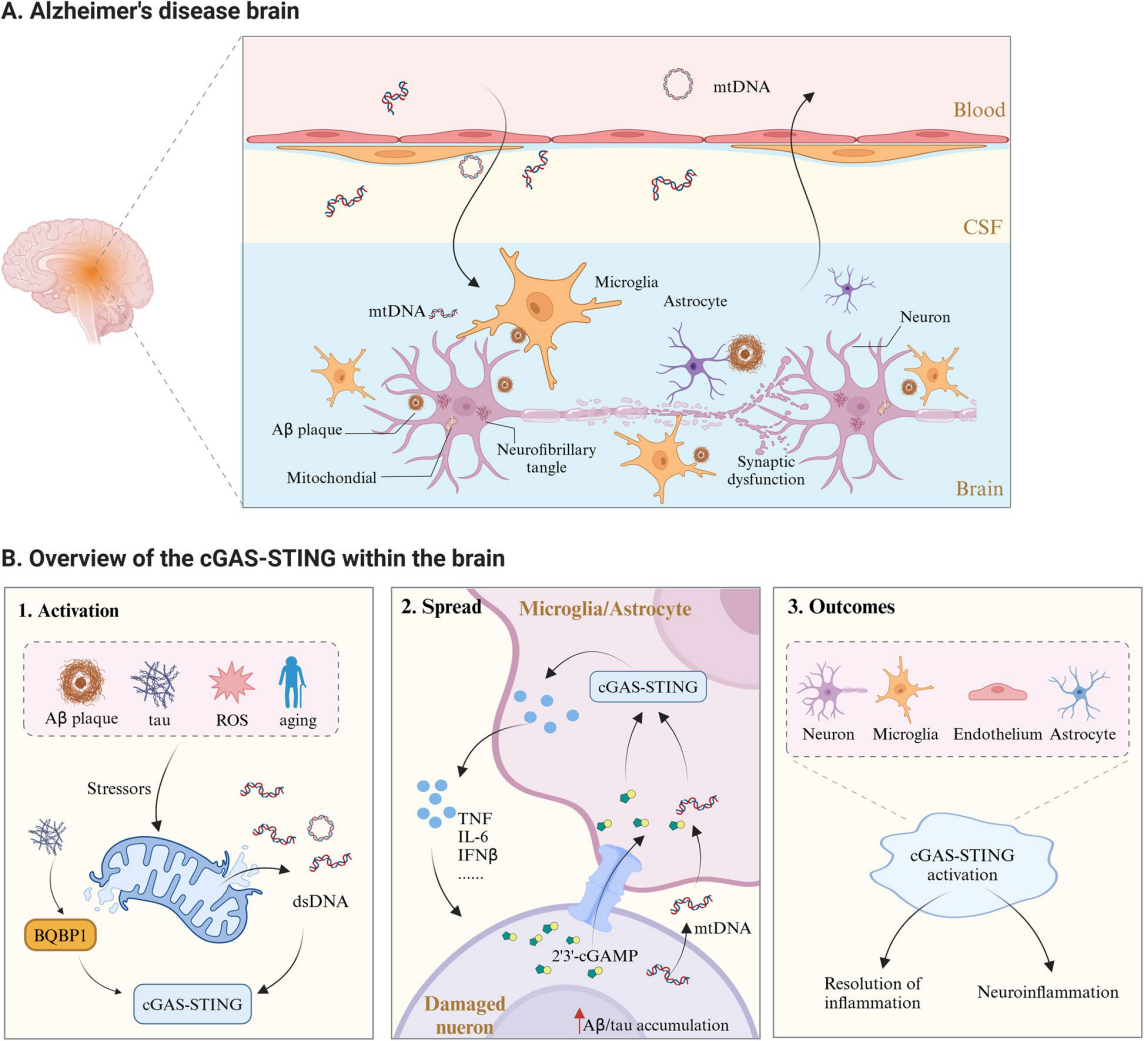
mtDNA-cGAS-STING signal pathway and neuroinflammation in AD. **A**. In the AD brain, the neurotoxic effects of primary pathological proteins, such as Aβ plaques and neurofibrillary tangles. The overwhelmed mitochondria of injured neurons release mtDNA into the extramitochondrial space, activating immune cells and forming a neuroinflammatory microenvironment in the CNS. **B**. The activation of the mtDNA-cGAS-STING pathway in the brain of AD. Firstly, various adverse factors in AD, such as Aβ plaques, hyperphosphorylated tau, ROS, and aging, stimulate the release of mtDNA from mitochondria in neurons. This mtDNA, along with AD pathological proteins, initiates the cGAS-STING pathway through multiple mechanisms. Secondly, cGAMP spreads through gap junctions in neurons and various glial cells, leading to the release of a series of cytokines by activated glial cells, and modulating neuronal inflammation. Ultimately, the activation of cGAS-STING would lead to complex outcomes. Various cell types in the brain play a role in the activation of this pathway, which, on one hand, can alleviate brain inflammation to some extent, while, on the other hand, its excessive activation forms a widespread neuroinflammatory network, exacerbating brain damage and promoting disease progression

AD is a neurodegenerative disease with an insidious onset, caused by the interaction of genetic and environmental factors [110]. Despite extensive research, its pathogenesis remains elusive. Emerging evidence suggests that mitochondrial disorders and innate immune responses play crucial roles in the development of AD [7, 111]. Specifically, numerous adverse factors, including elevated levels of pathogenic proteins in AD, could destabilize the neuronal mitochondrial genome [112, 113], leading to mitochondrial dysfunction, ultimately triggering the release of disrupted mtDNA into the extramitochondrial space to initiate a series of immune responses [114]. These maladaptive neuroinflammatory activities in the CNS are generally considered to exacerbate pathological changes and accelerate the rate of AD-related cognitive decline [115, 116]. Furthermore, several AD-related risk genes have been implicated in inflammation. As described by He et al., among the 50 AD-related risk loci identified through genome-wide association analysis (GWAS), more than half were significantly enriched or specifically expressed in immune cells, particularly microglia and macrophages, such as Age and apolipoprotein E (*APOE*), triggering receptors expressed on myeloid cells 2 (*TREM2*), and ATP-binding cassette subfamily A (*ABCA*) [117]. More importantly, the cGAS-STING signaling, a pivotal DNA-sensing pathway, links cytoplasmic mtDNA to sterile inflammation and the body’s innate immune system, and its activation has been identified across multiple cell types in AD brains [118]. Overall, mtDNA-cGAS-STING activation in AD highlights the intricate interplay between mitochondrial dysfunction and neuroinflammation (Fig. 3). Elucidating the molecular mechanisms of this immune-inflammatory signaling pathway holds immense promise for developing novel therapeutic interventions to mitigate the devastating impact of AD on both individuals and society as a whole.

### mtDNA disturbances in AD

Endogenous neuroprotection and repair within the body rely on the health of the mtDNA, whose turbulence inevitably leads to various AD pathological changes, including the abnormal aggregation of pathological amyloid-β (Aβ) plaques [119, 120] and hyperphosphorylated tau protein [121], impaired synaptic transmission and plasticity [122], cholinergic dysfunction [123], and neuroinflammation [124]. For example, mtDNA mutations in mice lead to an increase in Aβ42 levels and Aβ42 plaque density, and the accumulation of amyloidosis is caused by a decrease in the content of Aβ clearance enzymes, rather than an increase in Aβ production [125]. This decrease in Aβ clearance is likely due to energy supply disruption connected to mtDNA damage. Consistently, Scheffler et al. confirmed that mtDNA polymorphisms, such as mutations affecting oxidative phosphorylation or the tricarboxylic acid cycle, can cause reduced ATP levels and ATP-driven microglial activity, resulting in increased Aβ aggregation [126]. These studies suggested that mtDNA disruption exacerbates AD pathology by impairing mitochondrial function. In turn, mtDNA is susceptible to attacks from these destructive changes, further exacerbating mitochondrial injury [127, 128]. Specifically, Aβ can be transported to mitochondria through the translocase of the outer membrane (TOM) and localize to the mitochondrial cristae [129, 130], impairing oxidative phosphorylation pathways, causing mtDNA oxidative damage and DNA double-strand breaks (DSBs) [131]. This vicious cycle between mitochondrial defects and AD pathological impairment [132, 133] accelerates the rate of cognitive decline.

Several AD-related studies have provided abundant evidence to indicate the importance of mtDNA disturbances, primarily oxidative damage, mutations, and methylation transfer [134]. In the brain tissue samples from APPswe/PS1dE9 (APP/PS1) transgenic mouse model and AD patients, expression levels of genes necessary to mitochondrial replication and energy metabolism, including the peroxisome proliferator-activated receptor gamma coactivator 1 alpha (*PGC-1α)*, mitochondrial transcription factor A (*TFAM)*, and neurogenic differentiation factor-6 (*NEUROD6*), are significantly downregulated [135, 136], indicating a pronounced mtDNA maintenance defect in AD. Similarly, the oxidized base level of mtDNA in the brains of postmortem AD individuals has been shown to be markedly higher than that in an age-matched control group, and is approximately tenfold that of nuclear DNA (nDNA) [137]. Additionally, Chang et al. found a greater frequency of mtDNA point mutations in the hippocampal region of patients with AD, whereas the mtDNA (4977) and deletion mutation did not increase [138]. However, Hamblet et al. indicated that mtDNA delta 4977 in the temporal lobe cortex of postmortem AD brains was elevated 6.5 folds [139]. Notably, lower levels of mtDNA mutations have also been observed in patients with AD [124], which may be related to neuronal death and loss during AD progression. In addition, the degree of mtDNA methylation in AD remains highly uncertain [140, 141], possibly due to methodological differences among studies. In conclusion, current research on mtDNA abnormalities in AD has not reached a consensus, and mtDNA disorders exhibit significant heterogeneity. In the future, it will be necessary to interpret the role of mtDNA in AD pathogenesis using improved detection techniques, increased sample sizes, and other feasible approaches.

Alterations in mtDNA content in AD remain controversial [142–144] (Table 1). Increased levels of cytoplasmic mtDNA have been observed in the brains of 5xFAD mice compared with age-matched wild-type mice [145]. In line with this, Laura et al. reported elevated mtDNA counts in the CSF of patients with AD compared to those in normal individuals, albeit with considerable inter-individual heterogeneity [146]. Nevertheless, some studies have documented a reduction in mtDNAcn in the brain of AD patients [143, 144], as well as decreased levels of free mtDNA in the CSF of patients with preclinical AD [147]. A possible explanation is reduced energy metabolism or abnormal mitochondrial function in AD, which is reflected by mtDNAcn to some extent. While most studies have indicated a close relationship between abnormal mtDNA levels and AD pathology, determining whether changes in mtDNA content are causally related to the onset of AD and the specific mechanisms by which mtDNA levels affect AD pathogenesis remain significant challenges.

**Table 1:** Changes in mtDNA levels in clinical studies of AD

Sample type	Tissue/Cell Type	Sample size	Country	Technique	Trend	Disease Stage	Reference
Brain	-	AD/control: 282/351	UK	Exome sequencing	Decrease	AD	[143]
Brain	Hippocampal pyramidal neurons	AD/control: 10/9	US	qPCR	Decrease	AD	[148]
Brain	Frontal cortex	AD/control: 12/7	Spain	RT-PCR	Decrease	AD	[149]
	Hippocampus and cerebellum				No change	AD	
Blood	-	AD/control: 17/11			No change	AD	
Brain	Frontal cortex	AD/control: 23/40	US	qRT-PCR	Decrease	AD	[150]
Brain	Dorsolateral prefrontal cortex	AD/Non AD: 251/116; 57/30	US	WGS	Decrease	AD	[144]
	Posterior cingulate cortex	AD/Non AD: 45/21			Decrease		
	Cerebellum	AD/Non AD: 141/101			No change	AD	
	Temporal cortex	AD/control: 89/51			Decrease	AD	
	Frontal pole	AD/control: 205/132			Decrease	AD	
Brain	Temporal cortex	AD/control: 10/10	Brazil	ddPCR	Decrease	AD	[151]
	Cerebellum	AD/control: 10/10			No change	AD	
Brain	CSF	Symptomatic AD/Asymptomatic patients at risk of AD: 13/10 (study cohort a), 20/9 (study cohort b), 17/19 (validation cohort)	Spain	qPCR; ddPCR	Decrease	Symptomatic AD	[147]
Brain	CSF	AD patients progressed faster/control: 30/49	Germany	ddPCR	Decrease	AD patients progressed faster	[152]
		AD patients progressed lower/control: 16/49			No change	AD patients progressed lower	
Brain	CSF	AD/MCI/Preclinical AD/control: 58/42/20/140	Spain	ddPCR	Increase	Preclinical AD, MCI and AD	[146]
Brain	Pyramidal neurons	AD/old control/young control: 27/12/8	-	In situ hybridization	Increase	AD	[153]
Blood	Leukocyte	AD/control: 82/82	Tianjin, China	qRT-PCR	Decrease	AD	[154]
Blood	CD4 + , CD19 + and CD56 + peripheral lymphocytes	Early-stage AD/late-stage AD/control: 30/30/30	Turkey	RT-PCR	Decrease	Early-and late-stage AD	[155]
	CD8 + peripheral lymphocytes			RT-PCR	Decrease	Late-stage AD	
Blood	Plasma	Cognitive impaired/control: 11/35	US	RT-PCR	Increase	Cognitive impaired	[156]
	Buffy coat				Decrease		
Brain	Parietal cortex	AD/MCI/control: 24/6/16	UK	qRT-PCR	Decrease	MCI and AD	[157]
Blood	Leukocyte	Cognitive impaired/control: 29/78	Korea	qPCR	Decrease	Cognitive impaired	[158]
Blood	Leukocyte	Cognitive dysfunction/control: 125/61	Korea	RT-PCR	Decrease	Cognitive dysfunction	[159]
Blood	whole blood	AD/MCI/control: 28/31/28	UK	qRT-PCR	No change	MCI and AD	[142]
Brain	Dorsolateral prefrontal cortex	-	US	WGS	Decrease	MCI and AD	[160]
Blood	Buffy coat	AD/MCI/control: 31/72/149 (Mexican American)	US	qPCR	Decrease	AD	[161]
		AD/MCI/control: 63/28/129 (Non-Mexican American)			Increase	MCI	
	Plasma	AD/MCI/control: 38/82/91 (Mexican American) AD/MCI/control: 67/34/142 (Non-Mexican American)			No change	MCI and AD	
Blood	Leukocyte	AD/control: 600/601	Taiwanes, China	RT-PCR	Decrease	AD	[162]
Brain	-	-	US	WGS	Decrease	AD	[163]
Brain	Frontal cortex	AD/control: 13/25	US	qRT-PCR	Decrease	AD	[164]
Blood	Peripheral blood mononuclear cell	AD/MCI/control: 20/24/30 (Spanish cohort), 248/70/276 (Italian cohort)	Spanish, Italian	RT-PCR	Decrease	MCI and AD	[165]

### Direct evidence of the activation of cGAS-STING in AD

Although studies on the relationship between the cGAS-STING pathway and AD are currently limited, evidence of the activation of this pathway has been found in patients with AD and multiple AD models (Table 2). Increased interactions between dsDNA, RNA–DNA hybrids, and cGAS have been observed in AD patient-derived iPSCs [166], 5xFAD mice [145], or human AD fibroblasts [167]. Ferecskó et al. discovered that the expression levels of STING in neurons and endothelial cells were significantly elevated in CNS tissues extracted from AD patients [168]. Consistently, the phosphorylation levels of STING, TBK1, p-65, and IRF3 were found to be upregulated in the prefrontal cortex of AD patients [145]. Further studies demonstrated that cGAS gene deletion in 5xFAD mice alleviated cognitive impairment, Aβ aggregation, neuroinflammation, and cholinergic neuron damage; STING inhibitors also sustainably ameliorated AD pathogenesis, which may be partially attributable to the inhibition of neurotoxic A1 astrocytes [117, 145, 167].

**Table 2:** Research evidence on the correlation between the cGAS-STING pathway and AD

Model	Human sample	cGAS-STING activity	dsDNA levels	Intervention	Changes in dsDNA, cGAS-STING after Intervention	Intervention effect	References
APP/PS1 mice; Aβ induced HMC3 human microglial cells	-	Upregulated cGAS, STING	Increased cytosolic mtDNA level	NAD^+^	Decreased cytoplasmic mtDNA, cGAS, STING levels	Reduced neuroinflammation; improved cognition impairment and synaptic plasticity; prevented cellular senescence; promoted the protective microglial phenotype	[167]
5xFAD mice	Hippocampal region of AD patients	Upregulated cGAS, STING	Increased cytosolic mtDNA level	*Cgas* − / − ; RU.521; H-151	Suppressed the activation of the cGAS-STING pathway	Alleviated cognitive impairment, Aβ pathology, neuroinflammation; inhibited neurotoxic A1 astrocytic phenotype in microglia	[145]
APP/PS1 mice	-	Upregulated cGAS, STING	Increased cytosolic dsDNA level	Tetrahydroxy stilbene glycoside	Decreased cGAS, STING; alleviated the accumulation of cytosolic DNA	Ameliorated neuroinflammation; improved cognition impairment; inhibited the polarization of microglia toward M1-type	[169]
5 × FAD mice	-	Upregulated cGAS, STING	-	Cgas deletion; H-151	-	Suppressed the formation of neurotoxic A1 astrocytes; decreased oligomeric Aβ-induced neuronal toxicity	[170]
5xFAD mice	AD patients	Upregulated cGAS, STING	-	Microglia-specific cGAS knockout	-	Alleviated Aβ-induced cognitive impairment; limited plaque formation; reduced the levels of dystrophic neurites	[117]
APP/PS1 mice; sporadic AD patients derived-human skin fibroblast cell line	-	Upregulated cGAS, STING	Increased mtDNA level	NR	Elevated the cGAS-STING was normalized; reduced cytoplasmic DNA levels	Increased mitophagy; reduced neuroinflammation and cellular senescence; improved cognition and behavior; promotes anti-inflammatory microglial polarization	[171]
-	Temporal lobe of AD patients	Impaired cGAS-STING-interferon signaling	Increased dsDNA damage	-	-	-	[172]
PLD3 deficient SH-SY5Y cells	-	Upregulated cGAS, STING	Increased mtDNA level in lysosomes and cytoplasm	APP knockout; H151	Lowered STING activation;	Normalized APP-CTF levels and cholesterol biosynthesis	[173]
-	AD patients, dapsone, and anti-AD drug users in Hansen subjects	-	-	Dapsone (cGAS-STING pathway inhibitor)	-	Prevented AD exacerbation	[174]
APP/PS1 mice	AD patients	Downregulated cGAS, STING	Increased cytosolic mtDNA level	Melatonin	Promoting the cGAS-STING signaling; Ameliorated cytosolic mtDNA accumulation	Ameliorated APP/PS1-induced changes in cardiac geometry and function, apoptosis, mitochondrial integrity, and mitophagy	[175]
AD patient derived-iPSCs	-	Upregulated cGAS, STING	Increased cytosolic RNA–DNA hybrids level	c-Jun inhibition	Rescue RNA–DNA hybrid formation and the cGAS-STING activation	Rescued neuronal death and the impaired neurogenesis phenotype in AD progenitors	[166]
Aβ induced microglia	-	Upregulated cGAS, STING	-	RU.521; C-176; IFITM3 Knockdown	Decreased cGAS, STING	Suppressed the M1-like polarization of microglia; reduced neuroinflammation	[176]
APP/PS1 mice; Aβ induced HT22, N2a and SK‐N‐BE cell lines	-	Upregulated STING	Increased mtDNA leakage	Knocking out STING	Decreased STING	Inhibited Aβ1–42 and mtDNA induced neuron degeneration and cognitive impairment	[177]
STZ-treated HT22 murine hippocampal neurons; ICV-STZ rats	-	Upregulated STING	-	Silibinin	Decreased STING	Promoted survival of STZ-treated HT22 cells; ameliorates the cognitive impairment and anxiety/depression-like behavior of ICV-STZ rats; reduces STING-mediated neuroinflammation	[178]
P301S transgenic mice; tau induced microglia	AD patients	Upregulated cGAS, STING	Increased cytosolic mtDNA level	*Cgas* loss; TDI-6570	-	Restored synaptic integrity, plasticity and memory	[103]
APP/PS1 mice	-	Downregulated STING	-	cGAMP	Upregulated the expression of STING	Ameliorates cognitive deficits; improved pathological changes; decreases Aβ plaque load and neuron apoptosis	[179]
App^NL−G−F^/hTau double-knock-in mice; *APOE* ε4 human iPSC-derived microglia; Primary microglia treated with Aβ and tau	-	Upregulated cGAS, STING	Increased cytosolic mtDNA level	H-151; SN-011; STING knockout	Decreased STING	Reduced a wide range of AD pathogenic features; reduced gliosis and cerebral inflammation; prevented memory loss	[180]
*APOE4-R47H* female tauopathy mice	-	Upregulated cGAS, STING	Increased cytosolic mtDNA level	-	-	-	[181]

The text lists the research evidence on the causal relationship or correlation between AD and the cGAS-STING pathway. *PLD3* Phospholipase D3, *STZ* Streptozotocin, *ICV* Intracranial volume, *iPSC* Induced pluripotent stem cell, *FAD* Familial Alzheimer’s disease, *APP/PS1* APPswe/PS1dE9, *NAD*^+^ Nicotinamide adenine dinucleotide, *NR* Nicotinamide riboside, *RU.521* One of the cGAS inhibitors, *H-151* One of the STING inhibitors, *C-176* One of the STING inhibitors, *IFITM3* Interferon-induced transmembrane protein 3

Interestingly, two studies based on the APP/PS1 mouse model revealed reduced activity of the cGAS-STING pathway, whereas most research reports its activation (Table 2). Wang et al. found that the expression levels of cGAS and STING were significantly downregulated in the cardiomyocytes of APP/PS1 mice [175], despite the large aggregation of cytoplasmic mtDNA. Another study also observed a downward trend in STING expression levels in the APP/PS1 model compared to wild-type mice, although the differences were insignificant [179]. These contradictory results may be potentially due to differences in genetic backgrounds, stages of disease progression, and specific experimental conditions. Findings on this pathway derived from studies based on the APP/PS1 model should be carefully evaluated. In addition, STING in the Golgi apparatus was found to be nearly depleted in AD patients in a study by Nelson et al. [172]. A possible reason for this may be the prolonged activation of the cGAS-STING in the brain tissue, which has exhausted related components, and such prolonged activation may be mediated by the accumulated mtDNA and other dsDNA. However, it remains unclear whether the prolonged activation of this pathway truly exists in AD. Additionally, the threshold dose of dsDNA that triggers neuroinflammation and cellular damage, as well as the activation effects of different types of dsDNA, has yet to be fully defined. Overall, these changes reflect the plasticity of the pathway under different conditions to some extent and the potential protective effects of modulating this pathway in AD. Based on the existing evidence, understanding the role of the cGAS-STING in AD requires considering the composite effects of these factors, and how to balance the pathway to develop more effective therapeutic strategies.

### Activation of cGAS-STING is associated with mtDNA in AD

Xie et al. previously linked the activation of the cGAS-STING pathway to mtDNA in AD. In their research, mtDNA and 2’3’-cGAMP levels in primary microglia, neurons, and astrocytes were found to be significantly increased following treatment with oligomeric human Aβ42 peptides [145]. Similarly, Wilkins et al. demonstrated that mitochondrial lysates or mtDNA injected into the mouse hippocampus could act as a DAMP to induce inflammatory responses and affect AD-related biomarkers [182]. Furthermore, Hou et al. found that nicotinamide riboside (NR) supplementation can rescue abnormal mitochondrial autophagy and reduce cytoplasmic DNA levels in the APP/PS1 mouse model, thereby reducing the cGAS perception of mtDNA [167]. In addition, Zhao et al. suggested that the mtDNA released by neurons activates the cGAS-STING pathway in adjacent microglia, astrocytes, and other neuronal cells [183], but further research is needed to confirm this opinion in AD. Overall, these studies provide preliminary evidence that extracellularly released mtDNA likely facilitates communication between various types of neuronal cells, promotes the propagation of neuroinflammation, and exacerbates the inflammatory microenvironment.

### The relationship between cGAS-STING and the core pathological mechanism of AD

Hyperactivation of cGAS-STING and the aggregation of Aβ proteins are adverse factors in AD, and their interactions accelerate the pathological progression of AD [176]. Acker et al. found that the activation of cGAS-STING upregulated autophagy and induced amyloid precursor protein-C-terminal fragment (APP-CTF) accumulation [173]. Furthermore, p-STING primarily colocalized with the activated microglia marker CD68 around Aβ plaques in 5 × FAD mice [145], which may be related to ER and mitochondrial stress induced by the accumulation of neurotoxic proteins. In contrast, inhibition of the cGAS-STING and its downstream signaling could significantly alleviate Aβ toxicity [89, 117, 173]. These studies emphasize the importance of the cGAS-STING pathway in the neurotoxic process induced by Aβ aggregation and enrich the pathogenic mechanisms of Aβ as well.

The interaction between cGAS-STING and aberrantly aggregated hyperphosphorylated tau protein also contributes to AD development. Sequencing results from hippocampal tissue in a tau-induced disease model revealed elevated levels of cGAS and STING, while genetic ablation of cGAS in mice with tauopathy alleviated the activation of IFN-I response in microglia, without altering the pathological tau load in the brain. Additionally, pharmacological inhibition using TDI-6570, a non-toxic, brain-penetrant cGAS inhibitor, enhanced the neuronal MEF2C transcriptional network in the tauopathy mouse model, restoring synaptic integrity, plasticity, and memory [103]. Another recent study demonstrated that pharmacological inhibition of STING significantly reduces various pathological tau phosphorylation events in the AppNL-G-F/hTau double-knockin mouse model, further supporting the role of the cGAS-STING pathway in tau pathologies [180]. These studies revealed the critical role of the cGAS-STING pathway in AD, especially in its interaction with Aβ and tau. The modulation of this pathway may reduce the deposition of these pathological proteins, inhibit neuroinflammation, and protect neuronal and synaptic functions.

### The relationship between the cGAS-STING pathway and APOE genotype

APOE ε4 is the strongest genetic risk factor for late-onset sporadic AD, exacerbating neurodegeneration induced by tau [184]. This process is partially attributed to a crucial immune hub, which involves the activation of the interferon pathway in microglia and interactions with cytotoxic T cells [185, 186]. Recent studies indicate that cGAS-STING plays a significant role in this context as well, further linking innate immunity to neuroinflammatory responses in AD. Chung et al. found that APOE ε4 allele, like Aβ and tau, upregulated cGAS and STING expression in microglia. The knockout or pharmacological inhibition of STING reversed the reactivity of APOE ε4 human iPSC-derived microglia, significantly reducing the Aβ burden and tau hyperphosphorylation while preserving memory functions in AppNL-G-F/hTau-dKI mice [180]. In addition, the R47H variant of the TREM2 and female sex are risk factors for sporadic AD. A study by Carling et al. found that APOE ε4 and R47H amplify the tau-induced microglial cGAS-STING-IFN-I responses in female mice, worsening neurodegeneration through upregulated cGAS and BAX-dependent microglial senescence, suggesting that enhanced IFN-I signaling under APOE ε4 and R47H backgrounds could be an important pathological mechanism in AD [181]. Specific APOE mutations, such as the R136S mutation on APOE3, could reduce tau-related pathological burden by inhibiting the cGAS-STING-IFN signaling pathway, and treatment with cGAS inhibitor prevented tau-induced synaptic loss in E3/P301S mice [185, 187]. These findings emphasize not only the importance of the APOE-activated cGAS-STING pathway in tau pathology but also the role of microglia in this inflammatory process. Modulating the cGAS-STING pathway may provide new strategies for developing therapies against AD, especially in carriers of the APOE ε4 allele.

### Neuroinflammation induced by cGAS-STING and AD

Neuroinflammation is another significant feature of AD pathogenesis, involving aberrant activation of the innate immune system [188]. This effect is to some extent dependent on the disruption of the cGAS-STING pathway in different cells within the brain, especially in immune cells [189]. Although several types of neural cells can express cGAS and STING, microglia have been found to be the primary contributors to this pathway [117, 190, 191], which partially explains why they have been identified as major participants in neuroinflammation. Overall, abnormal activation of the cGAS-STING axis in diverse neuroglial cells is significant in the neuroinflammatory and neurodegenerative processes, particularly in microglia. Notably, moderate activation of this pathway may exert neuroprotective effects by suppressing inflammation. Evidence suggests that cGAMP treatment could activate the STING-IRF3 pathway to upregulate TREM2 expression, alleviating cognitive deficits and pathological changes in APP/PS1 transgenetic mice [179]. Another research has reported that ganciclovir can exert anti-inflammatory effects by mediating low therapeutic levels of IFNs, and this effect is STING-dependent [192]. Thus, the effects of cGAS-STING pathway activation vary, potentially depending on the differences in cell types and the extent of its activation. However, how to appropriately activate this pathway to achieve similar protective effects remains unclear. Further research is required to explore the balance between the anti-inflammatory and pro-inflammatory responses of this pathway in AD to achieve optimal neuroprotection.

The cGAS-STING pathway primarily induces neuroinflammation by regulating the expression of ISGs in inflammatory diseases [193, 194], including AD. Although the basal level of ISG mRNA in the CNS is generally much lower than in peripheral tissues, the brain is highly sensitive to IFN effects [195, 196]. In AD mouse models, pathological Aβ protein leads to a significant increase in ISG mRNA expression levels in the brain, and selective IFN receptor blockade effectively reduces persistent microgliosis and synaptic loss. Recent studies have also found that innate immune stimulation in neurons can drive seeded tau aggregation through the IFN-I response [197]. In clinical AD patients, the IFN pathway is also markedly upregulated and significantly correlated with disease severity and complement activation [198]. Additionally, the IFN-I produced by cGAS-STING pathway can induce a series of inflammatory responses by binding to its respective receptors in microglia, astrocytes, and neurons [199]. Then, these activated microglia secrete cytokines and chemokines, such as IL-1 and TNF-α, causing neurotoxicity through communicating with neurons [200]. Concisely, the cGAS-STING-IFN-I axis serves as an important mediator of neuroinflammation, forming a sophisticated regulatory network that bridges neuroglia and neurons in the CNS, leading to neuronal loss, and ultimately accelerating the onset of AD.

### Interaction between Cellular Senescence and cGAS-STING in AD

The interplay between cGAS-STING and cellular senescence is considered another crucial pathological process that accelerates AD progression [201, 202]. A recent study has shown that aged microglia, compared to young microglia, exhibit an increased mtDNA burden in the cytosol. This triggers the activation of the cGAS-STING pathway and subsequent aging-related reactive microglial transcriptional states, suggesting that the cGAS-STING pathway is a driving force behind age-related inflammation [203]. Consistently, the accumulation of senescent cells, along with cGAS activation and STING homo-dimer formation, has been observed in various types of neural cells during aging [204–207]. In the APP/PS1 mouse model, cGAS and STING expression levels were higher in aged mice compared to younger mice [167]. After cGAS knockout in mouse embryonic fibroblast cells, the rate of cellular senescence significantly slowed and progressed toward immortalization [85]. Another research has shown that blocking cGAS or STING suppressed etoposide-induced cellular senescence [167]. As such, dsDNA released during aging signals through the cGAS-STING pathway, and targeting the cGAS-STING pathway holds considerable promise for slowing AD development by inhibiting the aging process.

One of the important mechanisms through which the interplay of the cGAS-STING pathway and cellular senescence contribute to AD is the activation of the senescence-associated secretory phenotype (SASP) [202], which includes a variety of cytokines, chemokines, and growth factors secreted by senescent cells [208, 209]. These SASPs can recruit immune cells and regulate their activity [207], altering intercellular communication levels, and thus accelerating the aging of senescent cells and their neighboring cells [210, 211]. During aging, the process by which the cGAS-STING induces SASP has been considered to involve LINE-1 (L1), a key component of sterile inflammation and a hallmark of aging. According to research by De Cecco et al., the transcriptional derepression of L1 activates IFN-I responses, sustaining SASP and exacerbating inflammatory senescent phenotypes during cellular senescence, and knocking out cGAS or STING suppresses SASP gene expression in late senescent cells [212]. Other studies have also shown that cGAS or STING inhibition downregulates SASP gene expression in both mouse and human cells [85, 213].

cGAS-STING-mediated senescence is also involved in the release of mtDNA. Specifically, mitochondria exhibit structural abnormalities and accumulate mtDNA in the cytoplasm in aged microglia, which subsequently triggers a cGAS-dependent inflammatory response [203]. Notably, Victorelli et al. discovered an intriguing phenomenon during cellular aging in which a subset of mitochondria undergoes a process called miMOMP, leading to mtDNA translocation into the cytosol to trigger cGAS-STING activation [33]. These findings suggested that cGAS-STING is a crucial molecular link between various types of DNA damage, mitochondrial dysfunction, and aging. More research is needed to fully understand the intricate relationships between them and the precise molecular mechanisms underlying these connections, thus providing valuable evidence for the development of more effective targeted drugs for AD.

### The involvement of cGAS-STING in AD depends on microglia

Microglia, traditionally regarded as the resident macrophages of the brain, are involved in various physiological functions, ranging from immune surveillance to synaptic pruning [214]. In AD, microglia play a dual role, that is closely related to their involvement in the metabolic processes of neurotoxic proteins such as Aβ. On one hand, they can release degrading enzymes to degrade Aβ and further clear them through phagocytosis [215]. On the other hand, they further serve as carriers to seed Aβ, promoting its pathological spread throughout the CNS [216]. In turn, Aβ accumulation within the brain leads to the phenotypic shift of microglia, releasing neuroinflammatory substances and further contributing to neurotoxicity [176, 217]. Currently, the aberrant activation of cGAS-STING in microglia is believed to ultimately lead to neuronal dysfunction [117]. Modulating the activity of this pathway in microglia may aid in balancing the neuroprotective and neurotoxic effects, thereby slowing the progression of AD.

In recent studies, microglia have been regarded as the primary contributors to the cGAS-STING activation in AD, which is largely attributed to the relatively high expression levels of these two proteins [103, 170, 218], despite controversies regarding their expression levels across diverse cell types. Although Aβ oligomers can trigger cGAS activation in many types of neural cells, a more specific STING-IFN response is triggered in microglia, rather than in neurons and astrocytes [145]. Additionally, moderate expression levels of cGAS in neurons and oligodendrocytes were observed in another study, with low levels in astrocytes and endothelial cells [117]. Overall, cGAS-STING is largely not limited to a single cell type but encompasses a spectrum of cellular responses within the brain during AD. In addition to focusing on the complex role of this pathway in microglia, further exploration is required to understand the specific mechanisms in different cell types and how these cells communicate with each other, thus collectively slowing the development of AD.

**Fig. 4:**
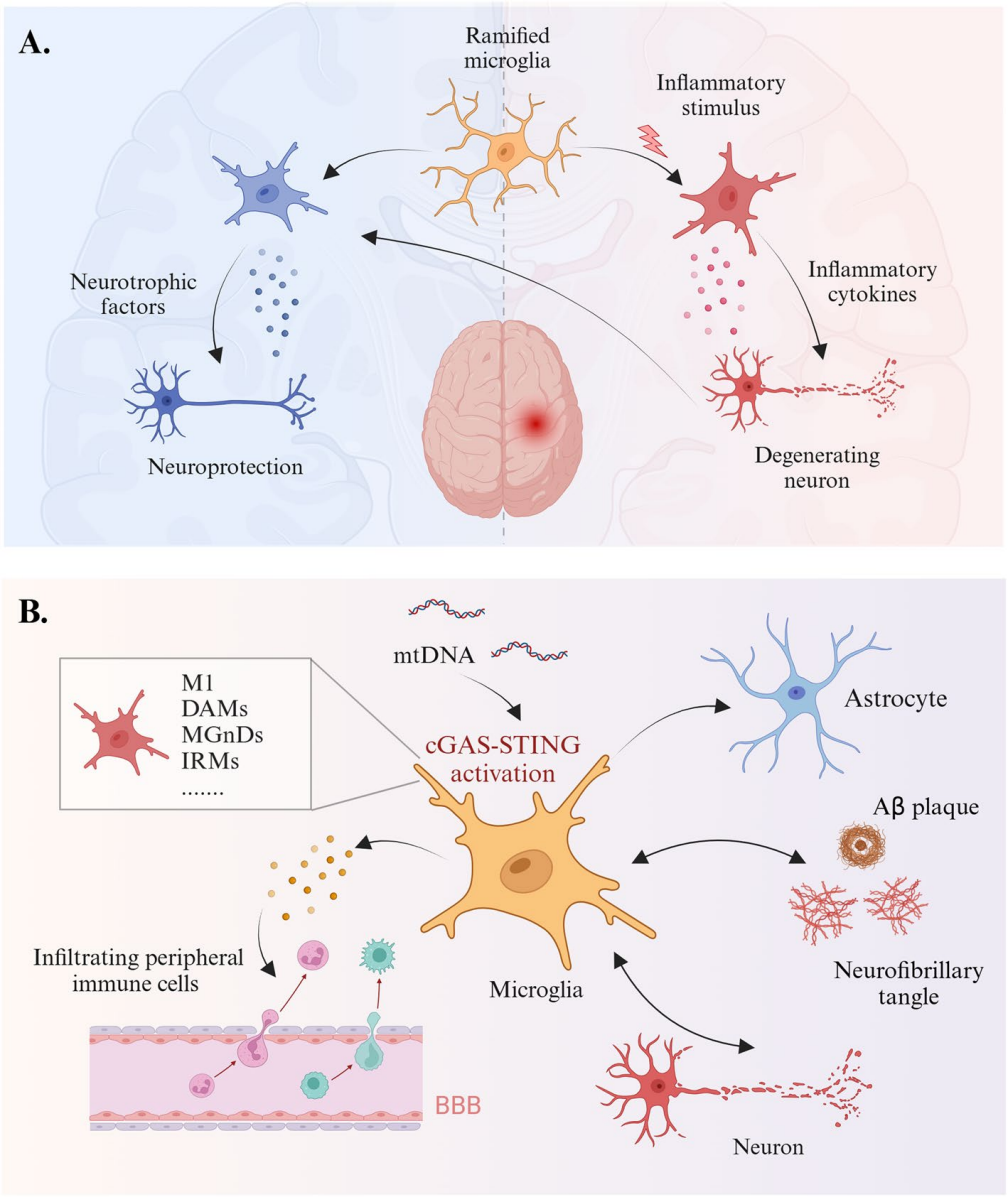
Microglia and the cGAS-STING pathway in AD. **A** The dual role of microglia in the CNS. Under physiological conditions, ramified microglia survey the brain microenvironment through their processes, sensing various damage signals. These highly dynamic cells perform immune surveillance, provide neurotrophic support, promote the establishment and maturation of neural circuits, and clear pathogens or debris, maintaining brain homeostasis. When the brain is challenged by pathogens or subjected to injury, activated microglia release various factors, including pro-inflammatory cytokines and chemokines. These factors help clear pathogens or toxins but may also lead to neuronal dysfunction and damage. **B** Activation of the cGAS-STING pathway in microglia in AD: Microglia are the primary cell type that activate cGAS-STING signaling in the brain. Pathological aggregates, such as Aβ plaques and tau proteins, cause mitochondrial damage and mtDNA release, leading to the activation of the cGAS-STING pathway in microglia, as well as astrocytes. Additionally, microglia secrete cytokines that induce the infiltration of peripheral immune cells, collectively modulating inflammation to alleviate neurodegeneration. However, overactivation of this system results in neuroinflammation, which can lead to neuronal dysfunction

Microglial neuroprotection or neurotoxicity depends on their phenotype and function to some extent (Fig. 4) [188], which can be regulated by the cGAS-STING pathway. AD-related research has focused mainly on traditional M1 and M2 microglial phenotypes currently [169, 176, 179], although evidence challenging this dichotomy exists. Single-nucleus RNA sequencing has identified other specific activation states in microglia related to cGAS, including interferon-responsive microglia (IRM), disease-associated microglia (DAM), and neurodegenerative microglia (MGnD). IRMs, a subset of microglia enriched with IFN response genes such as *Ifit*s, *Stat1*, *Sp100, Trim30a*, and *Parp14* [181, 203], play a crucial role in AD-related neuroinflammation. According to a recent study, IRMs are significantly enriched in APOE4-R47H tauopathy mice [181]. Another research found that the proportion of IRMs increases with age, a process further exacerbated by Aβ deposition [219]. Importantly, the phenotypic transition to IRMs is closely linked to DAMPs and the activation of the cGAS-STING pathway [220]. In the mg-*Cgas*^*R241E*^ microglial population, IFN signatures were broadly upregulated, indicating that cGAS activation induces microglia to transition into a distinct IFN-activated state, which in turn promotes neuroinflammation and aging processes [203]. The increase in DAM is another prominent feature in AD [221], and cGAS activation has been found to facilitate the transition of DAM subtypes from a low-activation state (DAM-1) to a high-activation state (DAM-2) [203]. In 5xFAD mice, the absence of cGAS significantly eliminates Aβ pathology-induced DAM markers [117]. However, these findings mainly rely on bulk transcriptomics and single-nucleus RNA sequencing, which may overlook the spatial differences in microglial activation states across different brain regions. The integration of spatial transcriptomics can better reveal the heterogeneity of cGAS-STING-driven microglial phenotypes across different brain regions in AD.

Various receptors expressed in microglia are linked to the activation of this pathway. Polyglutamine-binding protein 1 (PQBP1), an intracellular receptor capable of binding to HIV cDNA [222], has emerged as a participant in tau-mediated cGAS activation. Upon recognition of tau3R/4R, PQBP1 activates cGAS-STING, leading to the induction of NF-κB nuclear translocation. In contrast, depletion of PQBP1 markedly attenuates the recruitment of cGAS and NF-κB-dependent transcription of inflammation genes, thus mitigating inflammation in the brain and cognitive impairment [223]. As such, PQBP1 is a promising novel therapeutic target for AD and other tau proteinopathies. Another relevant receptor is TREM2, a transmembrane immune receptor predominantly expressed in microglia [224]. Xu et al. demonstrated that stimulation of the cGAMP-STING-IRF3 pathway can induce TREM2 expression, which promotes the phagocytosis of Aβ by microglia, and reduces Aβ deposition and neuronal loss, simultaneously attenuating the pathological morphology and cognitive deficits in AD. Therefore, upregulating TREM2 may exert a regulatory effect on the cGAS-STING pathway, potentially mitigating its neurotoxic effects to some extent.

The propagation of inflammatory signals triggered by STING-IFN in the microglia relies on intercellular gap junctions. Gap junction channels serve as vital conduits for intercellular communication, enabling the unrestricted flow of various small molecules and ions between neighboring cells [225]. Ablasser et al. demonstrated that the 2’3’-cGAMP produced by cGAS can exploit gap junctions to establish inflammatory crosstalk between human embryonic kidney cells and murine embryonic fibroblasts [226], and can also be transported between adjacent cells through specific anion channels [227]. In a mouse model induced by high-fat diets, the activation of cGAS-STING and microglia both occurred in the CNS; however, in their neuron-microglia co-culture system, a significant inhibition in cGAS-STING activation and inflammatory crosstalk was observed upon blocking gap junctions [228]. Accordingly, 2’3’-cGAMP produced in neurons can similarly converge into adjacent microglia, triggering the activation of STING-IFN and subsequently producing diverse inflammatory factors [229]. These inflammatory mediators not only stimulate microglia but can also influence other types of cells, such as astrocytes and neurons, thereby establishing a vast inflammatory microenvironment within the brain [230, 231]. The association between gap junctions and AD pathology described by Mei et al. is also noteworthy; in this study, they observed the enrichment of gap junction-forming proteins CX43 and CX30 near Aβ plaques, and blocking these gap junctions slowed pathological progression [232]. To conclude, gap junctions and other channels are likely involved in the exchange of cGAS-STING-mediated inflammatory signals between neurons and glial cells. Exploring the signal transduction mechanisms between neighboring neural cells may help to identify approaches to regulate this process of inflammatory communication, ultimately slowing down or even inhibiting the progression of AD.

## Potential therapeutic strategies and future directions

After mtDNA release, excessive activation of cGAS-STING and subsequent neuroinflammation inevitably lead to neuronal death and loss. Although the role of the mtDNA-cGAS-STING pathway in the CNS has only been partially revealed in recent years, its potential as an emerging target for AD treatment has gradually become apparent, and its significance and prospective utility cannot be ignored. Therefore, besides alleviating and inhibiting the abnormal release of mtDNA or other dsDNA caused by mitochondrial stress and autophagy imbalance [233], blocking the transmission of cGAS-STING signaling and the activation of its downstream inflammatory signals may represent a feasible approach for AD therapy [218].

**Fig. 5:**
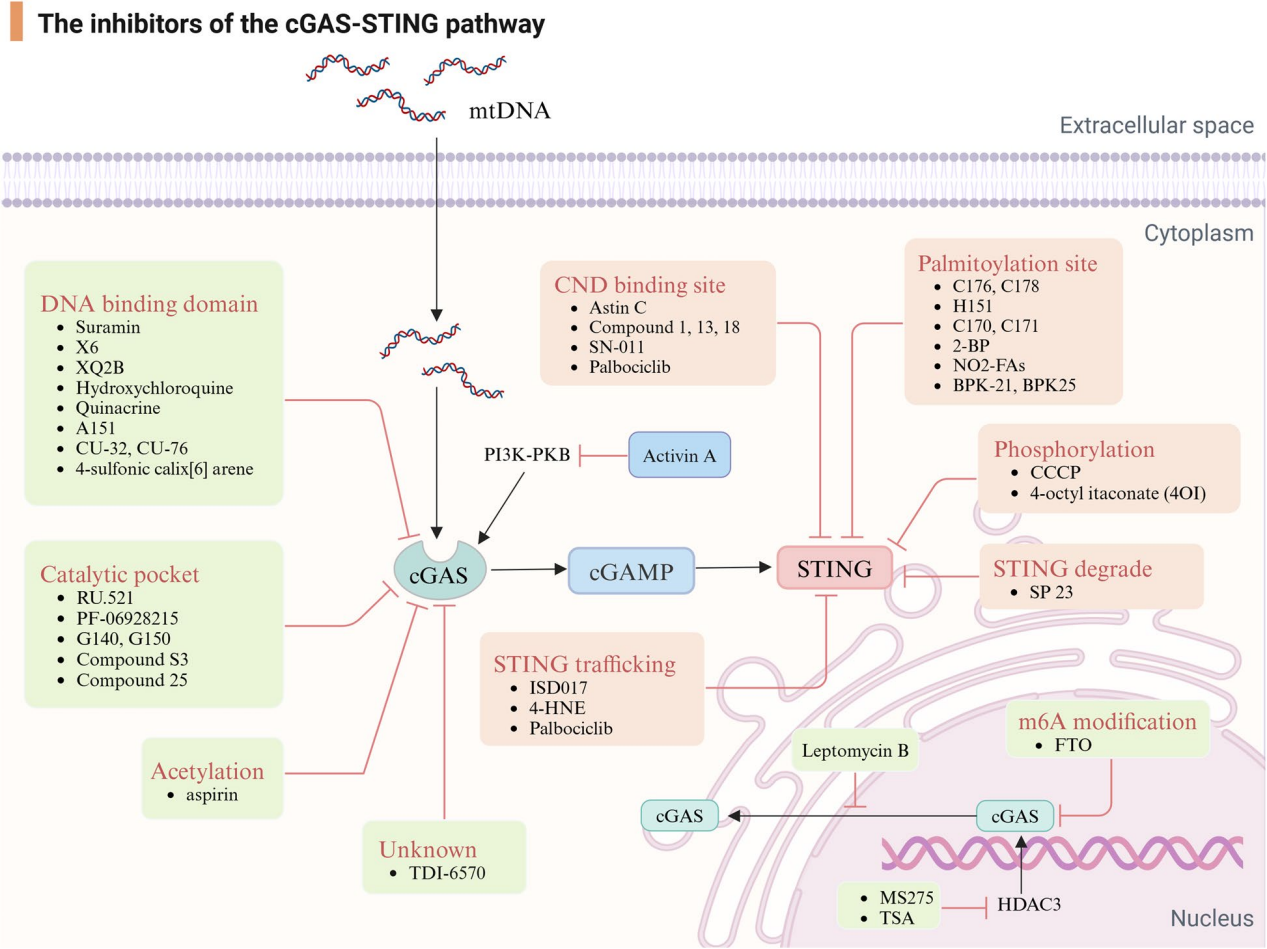
Overview of the inhibitors targeting the cGAS-STING signal pathway

Currently, a large body of preclinical research suggests that regulating the cGAS-STING is a promising intervention strategy (Table 3) (Fig. 5). Drugs targeting this pathway that have shown beneficial effects in AD animal or cellular models including H-151, RU.521, and C-176. For instance, H-151, inhibiting the palmitoylation of STING, and RU.521, targeting the catalytic site of cGAS, both significantly alleviated Aβ pathology in 5xFAD mice [145]; C-176 is an irreversible STING inhibitor that covalently binds to the Cys91 site of STING [234], significantly preventing neuroinflammation in microglia treated with Aβ_25–35_, and showing even more pronounced effects with combined treatment with RU.521 [176]. In addition, certain drugs, such as NR and its metabolite NAD^+^, the endogenous hormone melatonin, and the antibiotic dapsone [167, 171, 174, 175], all can non-specifically modulate this pathway to alleviate AD pathology. Although no drugs targeting this pathway have yet been approved for AD treatment, these studies provide valuable insights for the development of therapeutic drugs for AD in the future.

**Table 3:** The inhibitors targeting the cGAS-STING pathway

Targets	Agent	Mechanism	Pharmacological effect	References
cGAS Inhibitors	A151	Competes with DNA	Inhibits IFN-I production; attenuates brain inflammatory burden	[235, 236]
	Suramin	Displaces dsDNA from cGAS	Reduces IFN-I production	[237]
	AMDs (Hydroxychloroquine, Quinacrine, Chloroquine)	Disrupts the binding of dsDNA with cGAS	Reduces IFN-β production	[238, 239]
	X6	Disrupts the binding of dsDNA with cGAS	Reduces ISG expression and cGAMP production	[239]
	XQ2B	Targets the interface between cGAS and dsDNA and phase separation	Suppresses the elevated levels of type I interferon and proinflammatory cytokines	[240]
	CU.32, CU.76	Binds to the zinc-binding site of cGAS	Reduces IFN-β production	[241]
	4-sulfonic calix [6] arene	Influences both the dsDNA-binding site on cGAS and the 2′,3′-cGAMP	Dose-dependent inhibition in poly dA:dT-induced IFN-β release	[242]
	RU.521	Occupies the catalytic site of cGAS; reduce its affinity for ATP and GTP	Suppresses IFN- I production	[243]
	PF-06928215	Has high affinity for the catalytic site	Exhibits no inhibitory potency against dsDNA-induced IFN-β expression in cellular cGAS assays	[244]
	G140, G150	Inhibits the catalytic activity	Inhibits dsDNA-triggered interferon expression	[245]
	Compound S3	Inhibits the catalytic activity	-	[246]
	Compound 25	Occupies the catalytic site of cGAS	Dramatically suppresses the dsDNA-induced phosphorylation of the downstream STING/TBK1/IRF3 signaling and the mRNA expression of the downstream ISGs	[247, 248]
	EGCG	Disrupts cGAS activation by inhibiting of G3BP1	Inhibits self DNA-induced autoinflammatory responses and ISG expression	[249]
	aspirin	Inhibits cGAS by enforcing its acetylation	Suppresses self-DNA-induced autoimmunity in AGS patient cells and in an AGS mouse model	[194]
	FTO	Decreases cGAS mRNA stability by inhibition of m6A modification	Significantly alleviated brain injury and microglia-mediated inflammatory response	[250]
	Leptomycin B	Blocks the nuclear export of cGAS	Inhibits the expression of IFN-β and ISG mRNA	[251]
	Entinostat (MS275)	Suppresses cGAS transcription by inhibiting HDAC3	Reduces IFN-β production	[109]
	Trichostatin A (TSA)	Suppresses cGAS transcription by inhibiting HDAC3	Reduces IFN-β production	[109]
	Activin A	Inhibits cGAS-STING-mediated autophagy by the PI3K-PKB pathway	Alleviates neuronal injury	[252]
	Perillaldehyde	Inhibits cGAS activity through unknown mechanism	Reduction in type-I IFN- mediated inflammation in a mouse model of Aicardi-Goutières syndrome (AGS)	[253]
	TDI-6570	-	Suppresses IFN- I production	[103]
STING Inhibitors	Astin C	Inhibits STING by targeting its C-terminal activation pocket	Inhibits cytosolic DNA-trigged gene expression	[254]
	Compound 18	Inhibits STING by competing with cGAMP	Functional inhibition of STING-mediated cytokine release	[255]
	SN-011	Suppresses STING activation by blocking CDN binding pocket	Inhibits interferon and inflammatory cytokine induction induced by 2′3′-cGAMP	[256]
	C-176, C-178	Inhibits the palmitoylation of STING	Strongly reduced STING-mediated IFNβ reporter activity	[234]
	C-170, C-171, H-151	Inhibits the palmitoylation of STING	Abrogation of type I IFN responses, reduction of TBK1 phosphorylation and systemic cytokine responses	[234]
	NO2-FAs	Blocks STING Palmitoylation	Inhibit Release of Type I IFN	[257]
	2-bromopalmitate (2-BP)	Inhibits the palmitoylation of STING	Abolishes the type I interferon response	[258]
	BPK-21, BPK-25	Inhibits the palmitoylation of STING	-	[259]
	CCCP	Inhibits phosphorylation of STING and the interaction between STING and TBK-1	Inhibits STING-mediated IFN-β production	[260]
	4-octyl itaconate (4OI)	Inhibits phosphorylation of STING by alkylating its cysteine sites 65, 71, 88	Represses type I IFN production	[261]
	ISD017	Blocks the essential trafficking of STING from the ER to Golgi	Inhibits the expression of IFN-I and blocks pathological cytokine responses	[262]
	4-HNE	Inhibits STING translocation from the ER to the Golgi	-	[263]
	Palbociclib	Targets Y167 of STING to block its dimerization	Alleviates autoinflammation	[264]
	SP 23	Modulates STING-degrading activities	Reduces proinflammatory cytokines and inhibited the expression of p-TBK1	[265]

Despite significant progress in the research on cGAS-STING and its related modulators, there are still some issues that require clarification. Firstly, the cGAS and STING proteins are widely expressed in various tissues and cells, which may lead to significant adverse reactions when cGAS and STING modulators are used. Thus, when developing drugs targeting this pathway, it would be necessary to chemically optimize them to enhance target selectivity and deliver them selectively to the CNS, minimizing adverse reactions caused by their effects on the immune system throughout the body as much as possible. Additionally, owing to the functional diversity of cGAS and STING, and the complexity of the immune surveillance network [266, 267], complete inhibition of cGAS-STING signaling may not be a feasible approach. Specifically, prolonged inhibition of this pathway may be detrimental to the treatment of diseases requiring acute and beneficial initial neuroinflammatory responses, including stroke, spinal cord injury, and traumatic brain injury [268], although it may have relatively beneficial effects on chronic neurodegenerative diseases. At the same time, STING inhibitors may lead to excessive suppression of the body’s immune system, thus impairing the immune surveillance of tumor cells to increase the risk of tumor occurrence [269], and weakening antiviral and antibacterial responses that could result in severe infections [189]. It is also essential to identify the balance point for the regulation of this pathway to ensure the maximum alleviation of AD pathology while avoiding other adverse reactions.

Secondly, in studies related to AD treatment, the expression of cGAS and STING has been reported in multiple types of neural cells [103, 117]. However, whether this pathway mediates diverse pathophysiological responses in different cell types still remains unclear. Additionally, there is as yet no consensus regarding the cell types in the brain expressing cGAS and STING and their expression levels in different cell types; the intracellular localization of cGAS has not been fully elucidated as well [270]. Thus, necessitating further research to explore their cellular distribution to provide compelling evidence for more precise targeted therapy.

Furthermore, a comprehensive consideration of other influencing factors to improve drug treatment efficacy is equally important. In addition to cGAS, other DNA sensors, such as IFN-γ inducible protein 16 (IFI16) and DEAD-box helicase 41 (DDX41), have been found to mediate downstream signaling through STING and appear to complement cGAS in pathogen detection under certain circumstances [17, 271]. Among them, DDX41 modulates cGAS activation by regulating the homeostasis of dsDNA and ssDNA [272]. However, the mechanisms of interaction among these DNA sensors remain largely unclear. Simultaneous interference with these pathways could potentially impair immune surveillance and increase susceptibility to certain diseases. Another issue that requires attention is that existing research on this pathway is mostly based on animal or cell models, and there may be species differences in cGAS and STING [268], which could lead to poor clinical outcomes. In addition, drugs must be transported from the periphery to the CNS through the blood–brain barrier (BBB), increasing BBB permeability and realizing precise targeting therapy also needs to be given priority. However, current studies on these drugs primarily focus on their anti-inflammatory effects and are limited to peripheral administration in animal or in cell models, with insufficient research on their ability to cross the BBB, specific distribution, and metabolic processes within the CNS. Moreover, optimizing drug delivery systems, such as nanoparticle-based carriers, carrier/receptor-mediated endocytosis, and exosome-mediated drug delivery [273–276], may offer breakthroughs in targeting the cGAS-STING pathway for AD treatment.

## Conclusion

As research in the field has continued to advance, the mechanisms of mtDNA release and the specific role of the cGAS-STING pathway in AD are constantly being updated. Recent studies have revealed that mtDNA release and the activation of this pathway are interconnected events that collectively drive AD progression. Due to the extensive spectrum of pathogenic mechanisms formed by the interaction of this pathway with AD pathological processes, including neuroinflammation, aging, and neurodegeneration, further exploration of their causal relationships is essential. Importantly, the development of modulators targeting this pathway may provide new therapeutic strategies for AD. In summary, a thorough investigation into the role of the mtDNA-cGAS-STING pathway in AD is crucial for understanding its pathogenesis, identifying new therapeutic targets, and providing more possibilities for alleviating symptoms and slowing disease progression in patients with AD.

## Data Availability

Not applicable.

## Abbreviations

AD: Alzheimer’s disease
NFT: Neurofibrillary tangles
DAMPs: Damage-associated molecular patterns
PRRs: Pattern recognition receptors
cGAS: Cyclic GMP-AMP synthase
mtDNA: Mitochondrial DNA
dsDNA: Double-stranded DNA
STING: Stimulator of interferon genes
CNS: Central nervous system
PD: Parkinson’s disease
ALS: Amyotrophic lateral sclerosis
POCD: Postoperative cognitive dysfunction
TBE: Traumatic brain encephalopathy
miMOMP: Minority MOMP
VDAC: Voltage-dependent anion channel
OMM: Outer mitochondrial membrane
MOMP: Mitochondrial outer membrane permeabilization
EVs: Extracellular vesicles
NETs: Neutrophil extracellular traps
IFN: Interferon
IMM: Inner mitochondrial membrane
CICD: Caspase-independent cell death
MIMP: Mitochondrial inner membrane permeabilization
mt-dsRNA: Mitochondrial double-stranded RNA
ANT: Adenine nucleotide translocator
mPTP: Mitochondrial permeability transition pore
VRK2: Virus-related kinase 2
HK2: Hexokinase 2
ER: Endoplasmic reticulum
GSDMs: Gasdermins
IL-1β: Interleukin-1β
GSDMD: Gasdermin D
CSF: Cerebrospinal fluid
cf-mtDNA: Cell-free mtDNA
mtDNAcn: MtDNA copy numbers
TLR-9: Toll-like receptor-9
NLRP3: Nucleotide-binding oligomerization domain (NOD), leucine-rich repeat (LRR), and pyrin domain-containing protein 3
Mab21: Male abnormal gene family 21
2 ’3’-cGAMP: 2’3’-Cyclic GMP-AMP
GTP: Guanosine triphosphate
ATP: Adenosine triphosphate
CCT: C-terminal tail
TBK1: TANK-binding kinase 1
IRF3: Interferon regulatory factor 3
IFN-I: Type I interferons
ISGs: Interferon-stimulated genes
IKK: IκB kinase
NF-κb: Nuclear factor kappa-B
TNF-α: Tumor necrosis factor-alpha
IL-6: Interleukin-6
ERGIC: ER-Golgi intermediate compartment
LC3: Microtubule-associated protein 1 light chain 3
MEF2C: Myocyte enhancer factor 2c
TDP-43: Transactive response DNA binding protein of 43 kDa
GWAS: Genome-wide association analysis
APOE: Age and apolipoprotein E
TREM2: Triggering receptors expressed on myeloid cells 2
ABCA: ATP-binding cassette subfamily A
Aβ: β-Amyloid
TOM: Translocase of the outer membrane
DSBs: DNA double-strand breaks
APP/PS1: APPswe/PS1dE9
PGC-1α: Peroxisome proliferator-activated receptor gamma coactivator 1 alpha
TFAM: Mitochondrial transcription factor A
NEUROD6: Neurogenic differentiation factor-6
APP-CTF: Amyloid precursor protein-C-terminal fragment
SASP: Senescence-associated secretory phenotype
L1: LINE-1
IRM: Interferon-responsive microglia
DAM: Disease-associated microglia
MGnD: Neurodegenerative microglia
PQBP1: Polyglutamine-binding protein 1
IFI16: Interferon inducible protein 16
DDX41: DEAD-box helicase 41
BBB: Blood-brain barrier

## References

[1] Mangialasche F, Solomon A, Winblad B, Mecocci P, Kivipelto M. Alzheimer’s disease: clinical trials and drug development. Lancet Neurol. 2010;9:702–16. 20610346 10.1016/S1474-4422(10)70119-8

[2] DeTure MA, Dickson DW. The neuropathological diagnosis of Alzheimer’s disease. Mol Neurodegener. 2019;14:32. 31375134 10.1186/s13024-019-0333-5 PMC6679484

[3] Jia L, Du Y, Chu L, Zhang Z, Li F, Lyu D, et al. Prevalence, risk factors, and management of dementia and mild cognitive impairment in adults aged 60 years or older in China: a cross-sectional study. Lancet Public Health. 2020;5:e661–71. 33271079 10.1016/S2468-2667(20)30185-7

[4] Scheltens P, De Strooper B, Kivipelto M, Holstege H, Chételat G, Teunissen CE, et al. Alzheimer’s disease. Lancet. 2021;397:1577–90. 33667416 10.1016/S0140-6736(20)32205-4 PMC8354300

[5] Alzheimer’s disease facts and figures. Alzheimers Dement. 2023;19:1598–695. 36918389 10.1002/alz.13016

[6] Busche MA, Hyman BT. Synergy between amyloid-β and tau in Alzheimer’s disease. Nat Neurosci. 2020;23:1183–93. 32778792 10.1038/s41593-020-0687-6 PMC11831977

[7] Self WK, Holtzman DM. Emerging diagnostics and therapeutics for Alzheimer disease. Nat Med. 2023;29:2187–99. 37667136 10.1038/s41591-023-02505-2

[8] Munoz DG, Feldman H. Causes of Alzheimer’s disease. CMAJ. 2000;162:65–72. 11216203 PMC1232234

[9] Monteiro AR, Barbosa DJ, Remião F, Silva R. Alzheimer’s disease: Insights and new prospects in disease pathophysiology, biomarkers and disease-modifying drugs. Biochem Pharmacol. 2023;211: 115522. 36996971 10.1016/j.bcp.2023.115522

[10] van Olst L, Kamermans A, Halters S, van der Pol SMA, Rodriguez E, Verberk IMW, et al. Adaptive immune changes associate with clinical progression of Alzheimer’s disease. Mol Neurodegener. 2024;19:38. 38658964 10.1186/s13024-024-00726-8 PMC11044380

[11] Chen X, Holtzman DM. Emerging roles of innate and adaptive immunity in Alzheimer’s disease. Immunity. 2022;55:2236–54. 36351425 10.1016/j.immuni.2022.10.016 PMC9772134

[12] Gong T, Liu L, Jiang W, Zhou R. DAMP-sensing receptors in sterile inflammation and inflammatory diseases. Nat Rev Immunol. 2020;20:95–112. 31558839 10.1038/s41577-019-0215-7

[13] Zindel J, Kubes P. DAMPs, PAMPs, and LAMPs in Immunity and Sterile Inflammation. Annu Rev Pathol. 2020;15:493–518. 31675482 10.1146/annurev-pathmechdis-012419-032847

[14] Chow J, Franz KM, Kagan JC. PRRs are watching you: Localization of innate sensing and signaling regulators. Virology. 2015;479–480:104–9. 25800355 10.1016/j.virol.2015.02.051 PMC4424080

[15] Ablasser A, Chen ZJ. cGAS in action: Expanding roles in immunity and inflammation. Science. 2019;363:eaat8657. 10.1126/science.aat8657 30846571

[16] Kim J, Kim H-S, Chung JH. Molecular mechanisms of mitochondrial DNA release and activation of the cGAS-STING pathway. Exp Mol Med. 2023;55:510–9. 36964253 10.1038/s12276-023-00965-7 PMC10037406

[17] Cheng Z, Dai T, He X, Zhang Z, Xie F, Wang S, et al. The interactions between cGAS-STING pathway and pathogens. Signal Transduct Target Ther. 2020;5:91. 32532954 10.1038/s41392-020-0198-7 PMC7293265

[18] Liu Y, Duan R, Li P, Zhang B, Liu Y. 3-N-butylphthalide attenuates neuroinflammation in rotenone-induced Parkinson’s disease models via the cGAS-STING pathway. Int J Immunopathol Pharmacol. 2024;38:3946320241229041. 38315064 10.1177/03946320241229041 PMC10846052

[19] Yu C-H, Davidson S, Harapas CR, Hilton JB, Mlodzianoski MJ, Laohamonthonkul P, et al. TDP-43 Triggers Mitochondrial DNA Release via mPTP to Activate cGAS/STING in ALS. Cell. 2020;183:636-649.e18. 33031745 10.1016/j.cell.2020.09.020 PMC7599077

[20] Yang N-S-Y, Zhong W-J, Sha H-X, Zhang C-Y, Jin L, Duan J-X, et al. mtDNA-cGAS-STING axis-dependent NLRP3 inflammasome activation contributes to postoperative cognitive dysfunction induced by sevoflurane in mice. Int J Biol Sci. 2024;20:1927–46. 10.7150/ijbs.91543 PMC10929193 38481801

[21] Fritsch LE, Ju J, Gudenschwager Basso EK, Soliman E, Paul S, Chen J, et al. Type I Interferon Response Is Mediated by NLRX1-cGAS-STING Signaling in Brain Injury. Front Mol Neurosci. 2022;15: 852243. 35283725 10.3389/fnmol.2022.852243 PMC8916033

[22] Gustafsson CM, Falkenberg M, Larsson N-G. Maintenance and Expression of Mammalian Mitochondrial DNA. Annu Rev Biochem. 2016;85:133–60. 27023847 10.1146/annurev-biochem-060815-014402

[23] Yan C, Duanmu X, Zeng L, Liu B, Song Z. Mitochondrial DNA: Distribution, Mutations, and Elimination. Cells. 2019;8:379. 31027297 10.3390/cells8040379 PMC6523345

[24] Wang B, Huang M, Shang D, Yan X, Zhao B, Zhang X. Mitochondrial Behavior in Axon Degeneration and Regeneration. Front Aging Neurosci. 2021;13: 650038. 33762926 10.3389/fnagi.2021.650038 PMC7982458

[25] De Gaetano A, Solodka K, Zanini G, Selleri V, Mattioli AV, Nasi M, et al. Molecular Mechanisms of mtDNA-Mediated Inflammation Cells. 2021;10:2898. 34831121 10.3390/cells10112898 PMC8616383

[26] Collins LV, Hajizadeh S, Holme E, Jonsson I-M, Tarkowski A. Endogenously oxidized mitochondrial DNA induces in vivo and in vitro inflammatory responses. J Leukoc Biol. 2004;75:995–1000. 14982943 10.1189/jlb.0703328

[27] Fang C, Wei X, Wei Y. Mitochondrial DNA in the regulation of innate immune responses. Protein Cell. 2016;7:11–6. 26498951 10.1007/s13238-015-0222-9 PMC4707157

[28] Picca A, Calvani R, Coelho-Junior HJ, Marzetti E. Cell Death and Inflammation: The Role of Mitochondria in Health and Disease. Cells. 2021;10:537. 33802550 10.3390/cells10030537 PMC7998762

[29] Ng MYW, Wai T, Simonsen A. Quality control of the mitochondrion. Dev Cell. 2021;56:881–905. 33662258 10.1016/j.devcel.2021.02.009

[30] Chapman J, Ng YS, Nicholls TJ. The Maintenance of Mitochondrial DNA Integrity and Dynamics by Mitochondrial Membranes. Life (Basel). 2020;10:164. 32858900 10.3390/life10090164 PMC7555930

[31] McArthur K, Whitehead LW, Heddleston JM, Li L, Padman BS, Oorschot V, et al. BAK/BAX macropores facilitate mitochondrial herniation and mtDNA efflux during apoptosis. Science. 2018;359:eaao6047. 10.1126/science.aao6047 29472455

[32] Riley JS, Quarato G, Cloix C, Lopez J, O’Prey J, Pearson M, et al. Mitochondrial inner membrane permeabilisation enables mtDNA release during apoptosis. EMBO J. 2018;37: e99238. 30049712 10.15252/embj.201899238 PMC6120664

[33] Victorelli S, Salmonowicz H, Chapman J, Martini H, Vizioli MG, Riley JS, et al. Apoptotic stress causes mtDNA release during senescence and drives the SASP. Nature. 2023;622:627–36. 37821702 10.1038/s41586-023-06621-4 PMC10584674

[34] Kim J, Gupta R, Blanco LP, Yang S, Shteinfer-Kuzmine A, Wang K, et al. VDAC oligomers form mitochondrial pores to release mtDNA fragments and promote lupus-like disease. Science. 2019;366:1531–6. 31857488 10.1126/science.aav4011 PMC8325171

[35] Yan J, Liu W, Feng F, Chen L. VDAC oligomer pores: A mechanism in disease triggered by mtDNA release. Cell Biol Int. 2020;44:2178–81. 32716117 10.1002/cbin.11427

[36] Pérez-Treviño P, Velásquez M, García N. Mechanisms of mitochondrial DNA escape and its relationship with different metabolic diseases. Biochim Biophys Acta Mol Basis Dis. 2020;1866: 165761. 32169503 10.1016/j.bbadis.2020.165761

[37] Jiang X, Jiang H, Shen Z, Wang X. Activation of mitochondrial protease OMA1 by Bax and Bak promotes cytochrome c release during apoptosis. Proc Natl Acad Sci U S A. 2014;111:14782–7. 25275009 10.1073/pnas.1417253111 PMC4205663

[38] Cosentino K, Hertlein V, Jenner A, Dellmann T, Gojkovic M, Peña-Blanco A, et al. The interplay between BAX and BAK tunes apoptotic pore growth to control mitochondrial-DNA-mediated inflammation. Mol Cell. 2022;82:933-949.e9. 35120587 10.1016/j.molcel.2022.01.008 PMC8901441

[39] Gillies LA, Du H, Peters B, Knudson CM, Newmeyer DD, Kuwana T. Visual and functional demonstration of growing Bax-induced pores in mitochondrial outer membranes. Mol Biol Cell. 2015;26:339–49. 25411335 10.1091/mbc.E13-11-0638 PMC4294680

[40] White MJ, McArthur K, Metcalf D, Lane RM, Cambier JC, Herold MJ, et al. Apoptotic caspases suppress mtDNA-induced STING-mediated type I IFN production. Cell. 2014;159:1549–62. 25525874 10.1016/j.cell.2014.11.036 PMC4520319

[41] Rongvaux A, Jackson R, Harman CCD, Li T, West AP, de Zoete MR, et al. Apoptotic caspases prevent the induction of type I interferons by mitochondrial DNA. Cell. 2014;159:1563–77. 25525875 10.1016/j.cell.2014.11.037 PMC4272443

[42] Newman LE, Shadel GS. Mitochondrial DNA Release in Innate Immune Signaling. Annu Rev Biochem. 2023;92:299–332. 37001140 10.1146/annurev-biochem-032620-104401 PMC11058562

[43] Yoon J, Kim S, Lee M, Kim Y. Mitochondrial nucleic acids in innate immunity and beyond. Exp Mol Med. 2023;55:2508–18. 38036728 10.1038/s12276-023-01121-x PMC10766607

[44] Dhir A, Dhir S, Borowski LS, Jimenez L, Teitell M, Rötig A, et al. Mitochondrial double-stranded RNA triggers antiviral signalling in humans. Nature. 2018;560:238–42. 30046113 10.1038/s41586-018-0363-0 PMC6570621

[45] Becker Y, Marcoux G, Allaeys I, Julien A-S, Loignon R-C, Benk-Fortin H, et al. Autoantibodies in Systemic Lupus Erythematosus Target Mitochondrial RNA. Front Immunol. 2019;10:1026. 31134086 10.3389/fimmu.2019.01026 PMC6524553

[46] Baines CP. The molecular composition of the mitochondrial permeability transition pore. J Mol Cell Cardiol. 2009;46:850–7. 19233198 10.1016/j.yjmcc.2009.02.007 PMC2683186

[47] Murphy E, Steenbergen C. Mechanisms underlying acute protection from cardiac ischemia-reperfusion injury. Physiol Rev. 2008;88:581–609. 18391174 10.1152/physrev.00024.2007 PMC3199571

[48] García N, García JJ, Correa F, Chávez E. The permeability transition pore as a pathway for the release of mitochondrial DNA. Life Sci. 2005;76:2873–80. 15808887 10.1016/j.lfs.2004.12.012

[49] García N, Chávez E. Mitochondrial DNA fragments released through the permeability transition pore correspond to specific gene size. Life Sci. 2007;81:1160–6. 17870132 10.1016/j.lfs.2007.08.019

[50] Halestrap AP, McStay GP, Clarke SJ. The permeability transition pore complex: another view. Biochimie. 2002;84:153–66. 12022946 10.1016/s0300-9084(02)01375-5

[51] Nezu J, Oku A, Jones MH, Shimane M. Identification of two novel human putative serine/threonine kinases, VRK1 and VRK2, with structural similarity to vaccinia virus B1R kinase. Genomics. 1997;45:327–31. 9344656 10.1006/geno.1997.4938

[52] Zhu H, Li Q, Zhao Y, Peng H, Guo L, Zhu J, et al. Vaccinia-related kinase 2 drives pancreatic cancer progression by protecting Plk1 from Chfr-mediated degradation. Oncogene. 2021;40:4663–74. 34140642 10.1038/s41388-021-01893-4

[53] He W-R, Cao L-B, Yang Y-L, Hua D, Hu M-M, Shu H-B. VRK2 is involved in the innate antiviral response by promoting mitostress-induced mtDNA release. Cell Mol Immunol. 2021;18:1186–96. 33785841 10.1038/s41423-021-00673-0 PMC8093274

[54] Baik SH, Ramanujan VK, Becker C, Fett S, Underhill DM, Wolf AJ. Hexokinase dissociation from mitochondria promotes oligomerization of VDAC that facilitates NLRP3 inflammasome assembly and activation. Sci Immunol. 2023;8:eade7652. 10.1126/sciimmunol.ade7652 PMC10360408 37327321

[55] Miao R, Jiang C, Chang WY, Zhang H, An J, Ho F, et al. Gasdermin D permeabilization of mitochondrial inner and outer membranes accelerates and enhances pyroptosis. Immunity. 2023;56:2523-2541.e8. 37924812 10.1016/j.immuni.2023.10.004 PMC10872579

[56] Ding J, Wang K, Liu W, She Y, Sun Q, Shi J, et al. Pore-forming activity and structural autoinhibition of the gasdermin family. Nature. 2016;535:111–6. 27281216 10.1038/nature18590

[57] Monteleone M, Stanley AC, Chen KW, Brown DL, Bezbradica JS, von Pein JB, et al. Interleukin-1β Maturation Triggers Its Relocation to the Plasma Membrane for Gasdermin-D-Dependent and -Independent Secretion. Cell Rep. 2018;24:1425–33. 30089254 10.1016/j.celrep.2018.07.027

[58] Huang LS, Hong Z, Wu W, Xiong S, Zhong M, Gao X, et al. mtDNA Activates cGAS Signaling and Suppresses the YAP-Mediated Endothelial Cell Proliferation Program to Promote Inflammatory Injury. Immunity. 2020;52:475-486.e5. 32164878 10.1016/j.immuni.2020.02.002 PMC7266657

[59] Heilig R, Lee J, Tait SWG. Mitochondrial DNA in cell death and inflammation. Biochem Soc Trans. 2023;51:457–72. 36815695 10.1042/BST20221525 PMC9988000

[60] Miao N, Wang Z, Wang Q, Xie H, Yang N, Wang Y, et al. Oxidized mitochondrial DNA induces gasdermin D oligomerization in systemic lupus erythematosus. Nat Commun. 2023;14:872. 36797275 10.1038/s41467-023-36522-z PMC9935630

[61] Zhao C, Liang F, Ye M, Wu S, Qin Y, Zhao L, et al. GSDMD promotes neutrophil extracellular traps via mtDNA-cGAS-STING pathway during lung ischemia/reperfusion. Cell Death Discov. 2023;9:368. 37794018 10.1038/s41420-023-01663-z PMC10551007

[62] Deng X, Yang G, Zheng X, Yang Y, Qin H, Liu Z-X, et al. Plasma mtDNA copy numbers are associated with GSTK1 expression and inflammation in type 2 diabetes. Diabet Med. 2020;37:1874–8. 31502701 10.1111/dme.14132

[63] Liang Y, Fan S, Jiang Y, Ji T, Chen R, Xu Q, et al. Elevated serum mitochondrial DNA levels were associated with the progression and mortality in idiopathic pulmonary fibrosis. Int Immunopharmacol. 2023;123: 110754. 37573686 10.1016/j.intimp.2023.110754

[64] Leurs CE, Podlesniy P, Trullas R, Balk L, Steenwijk MD, Malekzadeh A, et al. Cerebrospinal fluid mtDNA concentration is elevated in multiple sclerosis disease and responds to treatment. Mult Scler. 2018;24:472–80. 28294696 10.1177/1352458517699874 PMC5987988

[65] Hajizadeh S, DeGroot J, TeKoppele JM, Tarkowski A, Collins LV. Extracellular mitochondrial DNA and oxidatively damaged DNA in synovial fluid of patients with rheumatoid arthritis. Arthritis Res Ther. 2003;5:R234-240. 12932286 10.1186/ar787 PMC193725

[66] Zhang Y, Liu X, Wiggins KL, Kurniansyah N, Guo X, Rodrigue AL, et al. Association of Mitochondrial DNA Copy Number With Brain MRI Markers and Cognitive Function: A Meta-analysis of Community-Based Cohorts. Neurology. 2023;100:e1930–43. 36927883 10.1212/WNL.0000000000207157 PMC10159770

[67] Chen S, Xie X, Wang Y, Gao Y, Xie X, Yang J, et al. Association between leukocyte mitochondrial DNA content and risk of coronary heart disease: a case-control study. Atherosclerosis. 2014;237:220–6. 25244506 10.1016/j.atherosclerosis.2014.08.051

[68] Stephens OR, Grant D, Frimel M, Wanner N, Yin M, Willard B, et al. Characterization and origins of cell-free mitochondria in healthy murine and human blood. Mitochondrion. 2020;54:102–12. 32781153 10.1016/j.mito.2020.08.002 PMC7508808

[69] Ding W, Chen J, Zhao L, Wu S, Chen X, Chen H. Mitochondrial DNA leakage triggers inflammation in age-related cardiovascular diseases. Front Cell Dev Biol. 2024;12:1287447. 38425502 10.3389/fcell.2024.1287447 PMC10902119

[70] Long G, Gong R, Wang Q, Zhang D, Huang C. Role of released mitochondrial DNA in acute lung injury. Front Immunol. 2022;13: 973089. 36059472 10.3389/fimmu.2022.973089 PMC9433898

[71] Civril F, Deimling T, de Oliveira Mann CC, Ablasser A, Moldt M, Witte G, et al. Structural mechanism of cytosolic DNA sensing by cGAS. Nature. 2013;498:332–7. 23722159 10.1038/nature12305 PMC3768140

[72] Bai J, Liu F. Nuclear cGAS: sequestration and beyond. Protein Cell. 2022;13:90–101. 34374004 10.1007/s13238-021-00869-0 PMC8783940

[73] Sun L, Wu J, Du F, Chen X, Chen ZJ. Cyclic GMP-AMP synthase is a cytosolic DNA sensor that activates the type I interferon pathway. Science. 2013;339:786–91. 23258413 10.1126/science.1232458 PMC3863629

[74] Herzner A-M, Hagmann CA, Goldeck M, Wolter S, Kübler K, Wittmann S, et al. Sequence-specific activation of the DNA sensor cGAS by Y-form DNA structures as found in primary HIV-1 cDNA. Nat Immunol. 2015;16:1025–33. 26343537 10.1038/ni.3267 PMC4669199

[75] Li X, Shu C, Yi G, Chaton CT, Shelton CL, Diao J, et al. Cyclic GMP-AMP synthase is activated by double-stranded DNA-induced oligomerization. Immunity. 2013;39:1019–31. 24332030 10.1016/j.immuni.2013.10.019 PMC3886715

[76] Crossley MP, Song C, Bocek MJ, Choi J-H, Kousouros JN, Sathirachinda A, et al. R-loop-derived cytoplasmic RNA-DNA hybrids activate an immune response. Nature. 2023;613:187–94. 36544021 10.1038/s41586-022-05545-9 PMC9949885

[77] Yu L, Liu P. Cytosolic DNA sensing by cGAS: regulation, function, and human diseases. Signal Transduct Target Ther. 2021;6:170. 33927185 10.1038/s41392-021-00554-y PMC8085147

[78] Chen Q, Sun L, Chen ZJ. Regulation and function of the cGAS-STING pathway of cytosolic DNA sensing. Nat Immunol. 2016;17:1142–9. 27648547 10.1038/ni.3558

[79] Gekara NO, Jiang H. The innate immune DNA sensor cGAS: A membrane, cytosolic, or nuclear protein? Sci Signal. 2019;12:eaax3521. 10.1126/scisignal.aax3521 31088977

[80] Barnett KC, Coronas-Serna JM, Zhou W, Ernandes MJ, Cao A, Kranzusch PJ, et al. Phosphoinositide Interactions Position cGAS at the Plasma Membrane to Ensure Efficient Distinction between Self- and Viral DNA. Cell. 2019;176:1432-1446.e11. 30827685 10.1016/j.cell.2019.01.049 PMC6697112

[81] Volkman HE, Cambier S, Gray EE, Stetson DB. Tight nuclear tethering of cGAS is essential for preventing autoreactivity. Elife. 2019;8: e47491. 31808743 10.7554/eLife.47491 PMC6927687

[82] Lu Y, Zhao M, Chen L, Wang Y, Liu T, Liu H. cGAS: action in the nucleus. Front Immunol. 2024;15:1380517. 38515746 10.3389/fimmu.2024.1380517 PMC10954897

[83] Li T, Huang T, Du M, Chen X, Du F, Ren J, et al. Phosphorylation and chromatin tethering prevent cGAS activation during mitosis. Science. 2021;371:eabc5386. 10.1126/science.abc5386 PMC8171060 33542149

[84] Zhong L, Hu M-M, Bian L-J, Liu Y, Chen Q, Shu H-B. Phosphorylation of cGAS by CDK1 impairs self-DNA sensing in mitosis. Cell Discov. 2020;6:26. 32351706 10.1038/s41421-020-0162-2 PMC7186227

[85] Yang H, Wang H, Ren J, Chen Q, Chen ZJ. cGAS is essential for cellular senescence. Proc Natl Acad Sci U S A. 2017;114:E4612–20. 28533362 10.1073/pnas.1705499114 PMC5468617

[86] Gao P, Ascano M, Wu Y, Barchet W, Gaffney BL, Zillinger T, et al. Cyclic [G(2’,5’)pA(3’,5’)p] is the metazoan second messenger produced by DNA-activated cyclic GMP-AMP synthase. Cell. 2013;153:1094–107. 23647843 10.1016/j.cell.2013.04.046 PMC4382009

[87] Xia P, Wang S, Gao P, Gao G, Fan Z. DNA sensor cGAS-mediated immune recognition. Protein Cell. 2016;7:777–91. 27696330 10.1007/s13238-016-0320-3 PMC5084157

[88] Ishikawa H, Barber GN. STING is an endoplasmic reticulum adaptor that facilitates innate immune signalling. Nature. 2008;455:674–8. 18724357 10.1038/nature07317 PMC2804933

[89] Chen K, Lai C, Su Y, Bao WD, Yang LN, Xu P-P, et al. cGAS-STING-mediated IFN-I Response in Host Defense and Neuroinflammatory Diseases. Curr Neuropharmacol. 2022;20:362–71. 34561985 10.2174/1570159X19666210924110144 PMC9413793

[90] Shang G, Zhang C, Chen ZJ, Bai X-C, Zhang X. Cryo-EM structures of STING reveal its mechanism of activation by cyclic GMP-AMP. Nature. 2019;567:389–93. 30842659 10.1038/s41586-019-0998-5 PMC6859894

[91] Zhang C, Shang G, Gui X, Zhang X, Bai X-C, Chen ZJ. Structural basis of STING binding with and phosphorylation by TBK1. Nature. 2019;567:394–8. 30842653 10.1038/s41586-019-1000-2 PMC6862768

[92] King KR, Aguirre AD, Ye Y-X, Sun Y, Roh JD, Ng RP, et al. IRF3 and type I interferons fuel a fatal response to myocardial infarction. Nat Med. 2017;23:1481–7. 29106401 10.1038/nm.4428 PMC6477926

[93] Abe T, Barber GN. Cytosolic-DNA-mediated, STING-dependent proinflammatory gene induction necessitates canonical NF-κB activation through TBK1. J Virol. 2014;88:5328–41. 24600004 10.1128/JVI.00037-14 PMC4019140

[94] Woznica A, Kumar A, Sturge CR, Xing C, King N, Pfeiffer JK. STING mediates immune responses in the closest living relatives of animals. Elife. 2021;10: e70436. 34730512 10.7554/eLife.70436 PMC8592570

[95] Gui X, Yang H, Li T, Tan X, Shi P, Li M, et al. Autophagy induction via STING trafficking is a primordial function of the cGAS pathway. Nature. 2019;567:262–6. 30842662 10.1038/s41586-019-1006-9 PMC9417302

[96] Yang J, Tang X, Nandakumar KS, Cheng K. Autophagy induced by STING, an unnoticed and primordial function of cGAS. Cell Mol Immunol. 2019;16:683–4. 31142798 10.1038/s41423-019-0240-2 PMC6804657

[97] Hu X, Zhang H, Zhang Q, Yao X, Ni W, Zhou K. Emerging role of STING signalling in CNS injury: inflammation, autophagy, necroptosis, ferroptosis and pyroptosis. J Neuroinflammation. 2022;19:242. 36195926 10.1186/s12974-022-02602-y PMC9531511

[98] Saitoh T, Fujita N, Hayashi T, Takahara K, Satoh T, Lee H, et al. Atg9a controls dsDNA-driven dynamic translocation of STING and the innate immune response. Proc Natl Acad Sci U S A. 2009;106:20842–6. 19926846 10.1073/pnas.0911267106 PMC2791563

[99] Liu D, Wu H, Wang C, Li Y, Tian H, Siraj S, et al. STING directly activates autophagy to tune the innate immune response. Cell Death Differ. 2019;26:1735–49. 30568238 10.1038/s41418-018-0251-z PMC6748081

[100] West AP, Khoury-Hanold W, Staron M, Tal MC, Pineda CM, Lang SM, et al. Mitochondrial DNA stress primes the antiviral innate immune response. Nature. 2015;520:553–7. 25642965 10.1038/nature14156 PMC4409480

[101] Zhang X, Wu J, Liu Q, Li X, Li S, Chen J, et al. mtDNA-STING pathway promotes necroptosis-dependent enterocyte injury in intestinal ischemia reperfusion. Cell Death Dis. 2020;11:1050. 33311495 10.1038/s41419-020-03239-6 PMC7732985

[102] Sliter DA, Martinez J, Hao L, Chen X, Sun N, Fischer TD, et al. Parkin and PINK1 mitigate STING-induced inflammation. Nature. 2018;561:258–62. 30135585 10.1038/s41586-018-0448-9 PMC7362342

[103] Udeochu JC, Amin S, Huang Y, Fan L, Torres ERS, Carling GK, et al. Tau activation of microglial cGAS-IFN reduces MEF2C-mediated cognitive resilience. Nat Neurosci. 2023;26:737–50. 37095396 10.1038/s41593-023-01315-6 PMC10166855

[104] Li Q, Yang L, Wang K, Chen Z, Liu H, Yang X, et al. Oxidized mitochondrial DNA activates the cGAS-STING pathway in the neuronal intrinsic immune system after brain ischemia-reperfusion injury. Neurotherapeutics. 2024;21: e00368. 38688786 10.1016/j.neurot.2024.e00368 PMC11284550

[105] Torralba D, Baixauli F, Villarroya-Beltri C, Fernández-Delgado I, Latorre-Pellicer A, Acín-Pérez R, et al. Priming of dendritic cells by DNA-containing extracellular vesicles from activated T cells through antigen-driven contacts. Nat Commun. 2018;9:2658. 29985392 10.1038/s41467-018-05077-9 PMC6037695

[106] Gambardella S, Limanaqi F, Ferese R, Biagioni F, Campopiano R, Centonze D, et al. ccf-mtDNA as a Potential Link Between the Brain and Immune System in Neuro-Immunological Disorders. Front Immunol. 2019;10:1064. 31143191 10.3389/fimmu.2019.01064 PMC6520662

[107] Liu Y, Zhang B, Duan R, Liu Y. Mitochondrial DNA Leakage and cGas/STING Pathway in Microglia: Crosstalk Between Neuroinflammation and Neurodegeneration. Neuroscience. 2024;548:1–8. 38685462 10.1016/j.neuroscience.2024.04.009

[108] Tresse E, Marturia-Navarro J, Sew WQG, Cisquella-Serra M, Jaberi E, Riera-Ponsati L, et al. Mitochondrial DNA damage triggers spread of Parkinson’s disease-like pathology. Mol Psychiatry. 2023;28:4902–14. 37779111 10.1038/s41380-023-02251-4 PMC10914608

[109] Liao Y, Cheng J, Kong X, Li S, Li X, Zhang M, et al. HDAC3 inhibition ameliorates ischemia/reperfusion-induced brain injury by regulating the microglial cGAS-STING pathway. Theranostics. 2020;10:9644–62. 32863951 10.7150/thno.47651 PMC7449914

[110] Guo T, Zhang D, Zeng Y, Huang TY, Xu H, Zhao Y. Molecular and cellular mechanisms underlying the pathogenesis of Alzheimer’s disease. Mol Neurodegener. 2020;15:40. 32677986 10.1186/s13024-020-00391-7 PMC7364557

[111] Ashleigh T, Swerdlow RH, Beal MF. The role of mitochondrial dysfunction in Alzheimer’s disease pathogenesis. Alzheimers Dement. 2023;19:333–42. 35522844 10.1002/alz.12683

[112] Reddy PH, Oliver DM. Amyloid Beta and Phosphorylated Tau-Induced Defective Autophagy and Mitophagy in Alzheimer’s Disease. Cells. 2019;8:488. 31121890 10.3390/cells8050488 PMC6562604

[113] Reddy PH, Yin X, Manczak M, Kumar S, Pradeepkiran JA, Vijayan M, et al. Mutant APP and amyloid beta-induced defective autophagy, mitophagy, mitochondrial structural and functional changes and synaptic damage in hippocampal neurons from Alzheimer’s disease. Hum Mol Genet. 2018;27:2502–16. 29701781 10.1093/hmg/ddy154 PMC6031001

[114] Wilkins HM, Carl SM, Weber SG, Ramanujan SA, Festoff BW, Linseman DA, et al. Mitochondrial lysates induce inflammation and Alzheimer’s disease-relevant changes in microglial and neuronal cells. J Alzheimers Dis. 2015;45:305–18. 25537010 10.3233/JAD-142334 PMC4605548

[115] VanItallie TB. Alzheimer’s disease: Innate immunity gone awry? Metabolism. 2017;69S:S41–9. 28129888 10.1016/j.metabol.2017.01.014

[116] Lecca D, Jung YJ, Scerba MT, Hwang I, Kim YK, Kim S, et al. Role of chronic neuroinflammation in neuroplasticity and cognitive function: A hypothesis. Alzheimers Dement. 2022;18:2327–40. 35234334 10.1002/alz.12610 PMC9437140

[117] He S, Li X, Mittra N, Bhattacharjee A, Wang H, Zhao S, et al. Microglial cGAS deletion protects against amyloid-β induced Alzheimer’s disease pathogenesis. bioRxiv. 2023;2023.08.07.552300.

[118] Li F, Wang N, Zheng Y, Luo Y, Zhang Y. cGAS- Stimulator of Interferon Genes Signaling in Central Nervous System Disorders. Aging Dis. 2021;12:1658–74. 34631213 10.14336/AD.2021.0304 PMC8460300

[119] Sorrentino V, Romani M, Mouchiroud L, Beck JS, Zhang H, D’Amico D, et al. Enhancing mitochondrial proteostasis reduces amyloid-β proteotoxicity. Nature. 2017;552:187–93. 29211722 10.1038/nature25143 PMC5730497

[120] Huang Z, Yan Q, Wang Y, Zou Q, Li J, Liu Z, et al. Role of Mitochondrial Dysfunction in the Pathology of Amyloid-β. J Alzheimers Dis. 2020;78:505–14. 33044180 10.3233/JAD-200519

[121] Weidling IW, Wilkins HM, Koppel SJ, Hutfles L, Wang X, Kalani A, et al. Mitochondrial DNA Manipulations Affect Tau Oligomerization. J Alzheimers Dis. 2020;77:149–63. 32804126 10.3233/JAD-200286 PMC7962146

[122] Verma H, Gangwar P, Yadav A, Yadav B, Rao R, Kaur S, et al. Understanding the neuronal synapse and challenges associated with the mitochondrial dysfunction in mild cognitive impairment and Alzheimer’s disease. Mitochondrion. 2023;73:19–29. 37708950 10.1016/j.mito.2023.09.003

[123] Bury AG, Pyle A, Elson JL, Greaves L, Morris CM, Hudson G, et al. Mitochondrial DNA changes in pedunculopontine cholinergic neurons in Parkinson disease. Ann Neurol. 2017;82:1016–21. 29149768 10.1002/ana.25099

[124] Shang D, Huang M, Wang B, Yan X, Wu Z, Zhang X. mtDNA Maintenance and Alterations in the Pathogenesis of Neurodegenerative Diseases. Curr Neuropharmacol. 2023;21:578–98. 35950246 10.2174/1570159X20666220810114644 PMC10207910

[125] Kukreja L, Kujoth GC, Prolla TA, Van Leuven F, Vassar R. Increased mtDNA mutations with aging promotes amyloid accumulation and brain atrophy in the APP/Ld transgenic mouse model of Alzheimer’s disease. Mol Neurodegener. 2014;9:16. 24885175 10.1186/1750-1326-9-16 PMC4028006

[126] Scheffler K, Krohn M, Dunkelmann T, Stenzel J, Miroux B, Ibrahim S, et al. Mitochondrial DNA polymorphisms specifically modify cerebral β-amyloid proteostasis. Acta Neuropathol. 2012;124:199–208. 22526016 10.1007/s00401-012-0980-x PMC3694593

[127] Mao P, Reddy PH. Aging and amyloid beta-induced oxidative DNA damage and mitochondrial dysfunction in Alzheimer’s disease: implications for early intervention and therapeutics. Biochim Biophys Acta. 2011;1812:1359–70. 21871956 10.1016/j.bbadis.2011.08.005 PMC3185172

[128] Gezen-Ak D, Yurttaş Z, Çamoǧlu T, Dursun E. Could Amyloid-β 1–42 or α-Synuclein Interact Directly with Mitochondrial DNA? A Hypothesis ACS Chem Neurosci. 2022;13:2803–12. 36125124 10.1021/acschemneuro.2c00512 PMC9542719

[129] Hansson Petersen CA, Alikhani N, Behbahani H, Wiehager B, Pavlov PF, Alafuzoff I, et al. The amyloid beta-peptide is imported into mitochondria via the TOM import machinery and localized to mitochondrial cristae. Proc Natl Acad Sci U S A. 2008;105:13145–50. 18757748 10.1073/pnas.0806192105 PMC2527349

[130] Devi L, Prabhu BM, Galati DF, Avadhani NG, Anandatheerthavarada HK. Accumulation of amyloid precursor protein in the mitochondrial import channels of human Alzheimer’s disease brain is associated with mitochondrial dysfunction. J Neurosci. 2006;26:9057–68. 16943564 10.1523/JNEUROSCI.1469-06.2006 PMC6675337

[131] Suberbielle E, Sanchez PE, Kravitz AV, Wang X, Ho K, Eilertson K, et al. Physiologic brain activity causes DNA double-strand breaks in neurons, with exacerbation by amyloid-β. Nat Neurosci. 2013;16:613–21. 23525040 10.1038/nn.3356 PMC3637871

[132] Mancuso M, Calsolaro V, Orsucci D, Siciliano G, Murri L. Is there a primary role of the mitochondrial genome in Alzheimer’s disease? J Bioenerg Biomembr. 2009;41:411–6. 19798559 10.1007/s10863-009-9239-1

[133] Oka S, Leon J, Sakumi K, Ide T, Kang D, LaFerla FM, et al. Human mitochondrial transcriptional factor A breaks the mitochondria-mediated vicious cycle in Alzheimer’s disease. Sci Rep. 2016;6:37889. 27897204 10.1038/srep37889 PMC5126576

[134] Antonyová V, Kejík Z, Brogyányi T, Kaplánek R, Pajková M, Talianová V, et al. Role of mtDNA disturbances in the pathogenesis of Alzheimer’s and Parkinson’s disease. DNA Repair (Amst). 2020;91–92: 102871. 32502755 10.1016/j.dnarep.2020.102871

[135] Pedrós I, Petrov D, Allgaier M, Sureda F, Barroso E, Beas-Zarate C, et al. Early alterations in energy metabolism in the hippocampus of APPswe/PS1dE9 mouse model of Alzheimer’s disease. Biochim Biophys Acta. 2014;1842:1556–66. 24887203 10.1016/j.bbadis.2014.05.025

[136] Hokama M, Oka S, Leon J, Ninomiya T, Honda H, Sasaki K, et al. Altered expression of diabetes-related genes in Alzheimer’s disease brains: the Hisayama study. Cereb Cortex. 2014;24:2476–88. 23595620 10.1093/cercor/bht101 PMC4128707

[137] Wang J, Xiong S, Xie C, Markesbery WR, Lovell MA. Increased oxidative damage in nuclear and mitochondrial DNA in Alzheimer’s disease. J Neurochem. 2005;93:953–62. 15857398 10.1111/j.1471-4159.2005.03053.x

[138] Chang SW, Zhang D, Chung HD, Zassenhaus HP. The frequency of point mutations in mitochondrial DNA is elevated in the Alzheimer’s brain. Biochem Biophys Res Commun. 2000;273:203–8. 10873587 10.1006/bbrc.2000.2885

[139] Hamblet NS, Castora FJ. Elevated levels of the Kearns-Sayre syndrome mitochondrial DNA deletion in temporal cortex of Alzheimer’s patients. Mutat Res. 1997;379:253–62. 9357554 10.1016/s0027-5107(97)00158-9

[140] Stoccoro A, Siciliano G, Migliore L, Coppedè F. Decreased Methylation of the Mitochondrial D-Loop Region in Late-Onset Alzheimer’s Disease. J Alzheimers Dis. 2017;59:559–64. 28655136 10.3233/JAD-170139

[141] Blanch M, Mosquera JL, Ansoleaga B, Ferrer I, Barrachina M. Altered Mitochondrial DNA Methylation Pattern in Alzheimer Disease-Related Pathology and in Parkinson Disease. Am J Pathol. 2016;186:385–97. 26776077 10.1016/j.ajpath.2015.10.004

[142] Lunnon K, Keohane A, Pidsley R, Newhouse S, Riddoch-Contreras J, Thubron EB, et al. Mitochondrial genes are altered in blood early in Alzheimer’s disease. Neurobiol Aging. 2017;53:36–47. 28208064 10.1016/j.neurobiolaging.2016.12.029

[143] Wei W, Keogh MJ, Wilson I, Coxhead J, Ryan S, Rollinson S, et al. Mitochondrial DNA point mutations and relative copy number in 1363 disease and control human brains. Acta Neuropathol Commun. 2017;5:13. 28153046 10.1186/s40478-016-0404-6 PMC5290662

[144] Klein H-U, Trumpff C, Yang H-S, Lee AJ, Picard M, Bennett DA, et al. Characterization of mitochondrial DNA quantity and quality in the human aged and Alzheimer’s disease brain. Mol Neurodegener. 2021;16:75. 34742335 10.1186/s13024-021-00495-8 PMC8572491

[145] Xie X, Ma G, Li X, Zhao J, Zhao Z, Zeng J. Activation of innate immune cGAS-STING pathway contributes to Alzheimer’s pathogenesis in 5×FAD mice. Nat Aging. 2023;3:202–12. 37118112 10.1038/s43587-022-00337-2

[146] Cervera-Carles L, Alcolea D, Estanga A, Ecay-Torres M, Izagirre A, Clerigué M, et al. Cerebrospinal fluid mitochondrial DNA in the Alzheimer’s disease continuum. Neurobiol Aging. 2017;53:192.e1-192.e4. 28089353 10.1016/j.neurobiolaging.2016.12.009

[147] Podlesniy P, Figueiro-Silva J, Llado A, Antonell A, Sanchez-Valle R, Alcolea D, et al. Low cerebrospinal fluid concentration of mitochondrial DNA in preclinical Alzheimer disease. Ann Neurol. 2013;74:655–68. 23794434 10.1002/ana.23955

[148] Rice AC, Keeney PM, Algarzae NK, Ladd AC, Thomas RR, Bennett JP. Mitochondrial DNA copy numbers in pyramidal neurons are decreased and mitochondrial biogenesis transcriptome signaling is disrupted in Alzheimer’s disease hippocampi. J Alzheimers Dis. 2014;40:319–30. 24448779 10.3233/JAD-131715

[149] Rodríguez-Santiago B, Casademont J, Nunes V. Is mitochondrial DNA depletion involved in Alzheimer’s disease? Eur J Hum Genet. 2001;9:279–85. 11313772 10.1038/sj.ejhg.5200629

[150] Coskun PE, Beal MF, Wallace DC. Alzheimer’s brains harbor somatic mtDNA control-region mutations that suppress mitochondrial transcription and replication. Proc Natl Acad Sci U S A. 2004;101:10726–31. 15247418 10.1073/pnas.0403649101 PMC490002

[151] Soltys DT, Pereira CPM, Rowies FT, Farfel JM, Grinberg LT, Suemoto CK, et al. Lower mitochondrial DNA content but not increased mutagenesis associates with decreased base excision repair activity in brains of AD subjects. Neurobiol Aging. 2019;73:161–70. 30359878 10.1016/j.neurobiolaging.2018.09.015

[152] Podlesniy P, Llorens F, Puigròs M, Serra N, Sepúlveda-Falla D, Schmidt C, et al. Cerebrospinal Fluid Mitochondrial DNA in Rapid and Slow Progressive Forms of Alzheimer’s Disease. Int J Mol Sci. 2020;21:6298. 32878083 10.3390/ijms21176298 PMC7503553

[153] Hirai K, Aliev G, Nunomura A, Fujioka H, Russell RL, Atwood CS, et al. Mitochondrial abnormalities in Alzheimer’s disease. J Neurosci. 2001;21:3017–23. 11312286 10.1523/JNEUROSCI.21-09-03017.2001 PMC6762571

[154] Lv X, Zhou D, Ge B, Chen H, Du Y, Liu S, et al. Association of Folate Metabolites and Mitochondrial Function in Peripheral Blood Cells in Alzheimer’s Disease: A Matched Case-Control Study. J Alzheimers Dis. 2019;70:1133–42. 31306134 10.3233/JAD-190477

[155] Terzioğlu G, Örmeci B, Türksoy Ö, Sayman C, Çınar N, Öztürk GA, et al. Mitochondrial depletion in CD4+ and CD19+ peripheral lymphocytes in early stage Alzheimer’s disease. Mech Ageing Dev. 2017;167:24–9. 28923392 10.1016/j.mad.2017.09.003

[156] Silzer T, Barber R, Sun J, Pathak G, Johnson L, O’Bryant S, et al. Circulating mitochondrial DNA: New indices of type 2 diabetes-related cognitive impairment in Mexican Americans. PLoS ONE. 2019;14: e0213527. 30861027 10.1371/journal.pone.0213527 PMC6414026

[157] Thubron EB, Rosa HS, Hodges A, Sivaprasad S, Francis PT, Pienaar IS, et al. Regional mitochondrial DNA and cell-type changes in post-mortem brains of non-diabetic Alzheimer’s disease are not present in diabetic Alzheimer’s disease. Sci Rep. 2019;9:11386. 31388037 10.1038/s41598-019-47783-4 PMC6684616

[158] Lee J-W, Park KD, Im J-A, Kim MY, Lee D-C. Mitochondrial DNA copy number in peripheral blood is associated with cognitive function in apparently healthy elderly women. Clin Chim Acta. 2010;411:592–6. 20114042 10.1016/j.cca.2010.01.024

[159] Lee J-Y, Kim J-H, Lee D-C. Combined Impact of Telomere Length and Mitochondrial DNA Copy Number on Cognitive Function in Community-Dwelling Very Old Adults. Dement Geriatr Cogn Disord. 2017;44:232–43. 28982094 10.1159/000480427

[160] Lynch MT, Taub MA, Farfel JM, Yang J, Abadir P, De Jager PL, et al. Evaluating genomic signatures of aging in brain tissue as it relates to Alzheimer’s disease. Sci Rep. 2023;13:14747. 37679407 10.1038/s41598-023-41400-1 PMC10484923

[161] Gorham IK, Reid DM, Sun J, Zhou Z, Barber RC, Phillips NR. Blood-Based mtDNA Quantification Indicates Population-Specific Differences Associated with Alzheimer’s Disease-Related Risk. J Alzheimers Dis. 2024;97:1407–19. 38250773 10.3233/JAD-230880 PMC11315371

[162] Liou C-W, Chen S-H, Lin T-K, Tsai M-H, Chang C-C. Oxidative Stress Biomarkers and Mitochondrial DNA Copy Number Associated with APOE4 Allele and Cholinesterase Inhibitor Therapy in Patients with Alzheimer’s Disease. Antioxidants (Basel). 2021;10:1971. 34943074 10.3390/antiox10121971 PMC8750673

[163] Harerimana NV, Paliwali D, Romero-Molina C, Bennett DA, Pa J, Goate A, et al. The role of mitochondrial genome abundance in Alzheimer’s disease. Alzheimers Dement. 2023;19:2069–83. 36224040 10.1002/alz.12812 PMC13043242

[164] Coskun PE, Wyrembak J, Derbereva O, Melkonian G, Doran E, Lott IT, et al. Systemic mitochondrial dysfunction and the etiology of Alzheimer’s disease and down syndrome dementia. J Alzheimers Dis. 2010;20(Suppl 2):S293-310. 20463402 10.3233/JAD-2010-100351 PMC4175722

[165] Delbarba A, Abate G, Prandelli C, Marziano M, Buizza L, Arce Varas N, et al. Mitochondrial Alterations in Peripheral Mononuclear Blood Cells from Alzheimer’s Disease and Mild Cognitive Impairment Patients. Oxid Med Cell Longev. 2016;2016:5923938. 26881032 10.1155/2016/5923938 PMC4736772

[166] Scopa C, Barnada SM, Cicardi ME, Singer M, Trotti D, Trizzino M. JUN upregulation drives aberrant transposable element mobilization, associated innate immune response, and impaired neurogenesis in Alzheimer’s disease. Nat Commun. 2023;14:8021. 38049398 10.1038/s41467-023-43728-8 PMC10696058

[167] Hou Y, Wei Y, Lautrup S, Yang B, Wang Y, Cordonnier S, et al. NAD+ supplementation reduces neuroinflammation and cell senescence in a transgenic mouse model of Alzheimer’s disease via cGAS-STING. Proc Natl Acad Sci U S A. 2021;118: e2011226118. 34497121 10.1073/pnas.2011226118 PMC8449423

[168] Ferecskó AS, Smallwood MJ, Moore A, Liddle C, Newcombe J, Holley J, et al. STING-Triggered CNS Inflammation in Human Neurodegenerative Diseases. Biomedicines. 2023;11:1375. 37239045 10.3390/biomedicines11051375 PMC10216406

[169] Gao D, Hao J-P, Li B-Y, Zheng C-C, Miao B-B, Zhang L, et al. Tetrahydroxy stilbene glycoside ameliorates neuroinflammation for Alzheimer’s disease via cGAS-STING. Eur J Pharmacol. 2023;953: 175809. 37328043 10.1016/j.ejphar.2023.175809

[170] Microglial cGAS-STING links innate immunity and Alzheimer’s disease. Nat Aging. 2023;3:155–6. 37118114 10.1038/s43587-022-00350-5

[171] Larrick JW, Mendelsohn AR. Modulation of cGAS-STING Pathway by Nicotinamide Riboside in Alzheimer’s Disease. Rejuvenation Res. 2021;24:397–402. 34694148 10.1089/rej.2021.0062

[172] Nelson TJ, Xu Y. Sting and p53 DNA repair pathways are compromised in Alzheimer’s disease. Sci Rep. 2023;13:8304. 37221295 10.1038/s41598-023-35533-6 PMC10206146

[173] Van Acker ZP, Perdok A, Hellemans R, North K, Vorsters I, Cappel C, et al. Phospholipase D3 degrades mitochondrial DNA to regulate nucleotide signaling and APP metabolism. Nat Commun. 2023;14:2847. 37225734 10.1038/s41467-023-38501-w PMC10209153

[174] Lee JH, Kanwar B, Lee CJ, Sergi C, Coleman MD. Dapsone is an anticatalysis for Alzheimer’s disease exacerbation. iScience. 2022;25:104274. 10.1016/j.isci.2022.104274 PMC9079171 35542045

[175] Wang S, Wang L, Qin X, Turdi S, Sun D, Culver B, et al. ALDH2 contributes to melatonin-induced protection against APP/PS1 mutation-prompted cardiac anomalies through cGAS-STING-TBK1-mediated regulation of mitophagy. Signal Transduct Target Ther. 2020;5:119. 32703954 10.1038/s41392-020-0171-5 PMC7378833

[176] Wu Z, Tang W, Ibrahim FEEM, Chen X, Yan H, Tao C, et al. Aβ Induces Neuroinflammation and Microglial M1 Polarization via cGAS-STING-IFITM3 Signaling Pathway in BV-2 Cells. Neurochem Res. 2023;48:2881–94. 37210413 10.1007/s11064-023-03945-5

[177] Xia X, He X, Zhao T, Yang J, Bi Z, Fu Q, et al. Inhibiting mtDNA-STING-NLRP3/IL-1β axis-mediated neutrophil infiltration protects neurons in Alzheimer’s disease. Cell Prolif. 2024;57: e13529. 37528567 10.1111/cpr.13529 PMC10771109

[178] Liu P, Chen W, Kang Y, Wang C, Wang X, Liu W, et al. Silibinin ameliorates STING-mediated neuroinflammation via downregulation of ferroptotic damage in a sporadic Alzheimer’s disease model. Arch Biochem Biophys. 2023;744: 109691. 37473980 10.1016/j.abb.2023.109691

[179] Xu Q, Xu W, Cheng H, Yuan H, Tan X. Efficacy and mechanism of cGAMP to suppress Alzheimer’s disease by elevating TREM2. Brain Behav Immun. 2019;81:495–508. 31283973 10.1016/j.bbi.2019.07.004

[180] Chung S, Jeong J-H, Park J-C, Han JW, Lee Y, Kim J-I, et al. Blockade of STING activation alleviates microglial dysfunction and a broad spectrum of Alzheimer’s disease pathologies. Exp Mol Med. 2024;56:1936–51. 39218977 10.1038/s12276-024-01295-y PMC11447230

[181] Carling GK, Fan L, Foxe NR, Norman K, Wong MY, Zhu D, et al. Alzheimer’s disease-linked risk alleles elevate microglial cGAS-associated senescence and neurodegeneration in a tauopathy model. Neuron. 2024;S0896–6273(24):00654–8. 10.1016/j.neuron.2024.09.006 PMC11624100 39353433

[182] Wilkins HM, Koppel SJ, Weidling IW, Roy N, Ryan LN, Stanford JA, et al. Extracellular Mitochondria and Mitochondrial Components Act as Damage-Associated Molecular Pattern Molecules in the Mouse Brain. J Neuroimmune Pharmacol. 2016;11:622–8. 27562848 10.1007/s11481-016-9704-7 PMC5097888

[183] Zhao Y, Liu B, Xu L, Yu S, Fu J, Wang J, et al. ROS-Induced mtDNA Release: The Emerging Messenger for Communication between Neurons and Innate Immune Cells during Neurodegenerative Disorder Progression. Antioxidants (Basel). 2021;10:1917. 34943020 10.3390/antiox10121917 PMC8750316

[184] Shi Y, Yamada K, Liddelow SA, Smith ST, Zhao L, Luo W, et al. ApoE4 markedly exacerbates tau-mediated neurodegeneration in a mouse model of tauopathy. Nature. 2017;549:523–7. 28959956 10.1038/nature24016 PMC5641217

[185] Nelson MR, Liu P, Agrawal A, Yip O, Blumenfeld J, Traglia M, et al. The APOE-R136S mutation protects against APOE4-driven Tau pathology, neurodegeneration and neuroinflammation. Nat Neurosci. 2023;26:2104–21. 37957317 10.1038/s41593-023-01480-8 PMC10689245

[186] Chen X, Firulyova M, Manis M, Herz J, Smirnov I, Aladyeva E, et al. Microglia-mediated T cell infiltration drives neurodegeneration in tauopathy. Nature. 2023;615:668–77. 36890231 10.1038/s41586-023-05788-0 PMC10258627

[187] Naguib S, Lopez-Lee C, Torres ER, Lee S-I, Zhu J, Zhu D, et al. APOE3 - R136S mutation confers resilience against tau pathology via cGAS-STING-IFN inhibition. bioRxiv. 2025;2024.04.25.591140. 10.1016/j.immuni.2025.05.023 PMC12406129 40555238

[188] Leng F, Edison P. Neuroinflammation and microglial activation in Alzheimer disease: where do we go from here? Nat Rev Neurol. 2021;17:157–72. 33318676 10.1038/s41582-020-00435-y

[189] Guo X, Yang L, Wang J, Wu Y, Li Y, Du L, et al. The cytosolic DNA-sensing cGAS-STING pathway in neurodegenerative diseases. CNS Neurosci Ther. 2024;30: e14671. 38459658 10.1111/cns.14671 PMC10924111

[190] Cox DJ, Field RH, Williams DG, Baran M, Bowie AG, Cunningham C, et al. DNA sensors are expressed in astrocytes and microglia in vitro and are upregulated during gliosis in neurodegenerative disease. Glia. 2015;63:812–25. 25627810 10.1002/glia.22786 PMC4657478

[191] Jeffries AM, Marriott I. Human microglia and astrocytes express cGAS-STING viral sensing components. Neurosci Lett. 2017;658:53–6. 28830822 10.1016/j.neulet.2017.08.039 PMC5645252

[192] Mathur V, Burai R, Vest RT, Bonanno LN, Lehallier B, Zardeneta ME, et al. Activation of the STING-Dependent Type I Interferon Response Reduces Microglial Reactivity and Neuroinflammation. Neuron. 2017;96:1290-1302.e6. 29268096 10.1016/j.neuron.2017.11.032 PMC5806703

[193] Reinert LS, Lopušná K, Winther H, Sun C, Thomsen MK, Nandakumar R, et al. Sensing of HSV-1 by the cGAS-STING pathway in microglia orchestrates antiviral defence in the CNS. Nat Commun. 2016;7:13348. 27830700 10.1038/ncomms13348 PMC5109551

[194] Dai J, Huang Y-J, He X, Zhao M, Wang X, Liu Z-S, et al. Acetylation Blocks cGAS Activity and Inhibits Self-DNA-Induced Autoimmunity. Cell. 2019;176:1447-1460.e14. 30799039 10.1016/j.cell.2019.01.016 PMC8274936

[195] Paul BD, Snyder SH, Bohr VA. Signaling by cGAS-STING in Neurodegeneration, Neuroinflammation, and Aging. Trends Neurosci. 2021;44:83–96. 33187730 10.1016/j.tins.2020.10.008 PMC8662531

[196] Ida-Hosonuma M, Iwasaki T, Yoshikawa T, Nagata N, Sato Y, Sata T, et al. The Alpha/Beta Interferon Response Controls Tissue Tropism and Pathogenicity of Poliovirus. J Virol. 2005;79:4460. 15767446 10.1128/JVI.79.7.4460-4469.2005 PMC1061561

[197] Sanford SAI, Miller LVC, Vaysburd M, Keeling S, Tuck BJ, Clark J, et al. The type-I interferon response potentiates seeded tau aggregation and exacerbates tau pathology. Alzheimers Dement. 2024;20:1013–25. 37849026 10.1002/alz.13493 PMC10916982

[198] Roy ER, Wang B, Wan Y, Chiu G, Cole A, Yin Z, et al. Type I interferon response drives neuroinflammation and synapse loss in Alzheimer disease. J Clin Investig. 2020;130:1912. 31917687 10.1172/JCI133737 PMC7108898

[199] Roy ER, Chiu G, Li S, Propson NE, Kanchi R, Wang B, et al. Concerted type I interferon signaling in microglia and neural cells promotes memory impairment associated with amyloid β plaques. Immunity. 2022;55:879-894.e6. 35443157 10.1016/j.immuni.2022.03.018 PMC9109419

[200] Harry GJ, Kraft AD. Neuroinflammation and microglia: considerations and approaches for neurotoxicity assessment. Expert Opin Drug Metab Toxicol. 2008;4:1265–77. 18798697 10.1517/17425255.4.10.1265 PMC2658618

[201] Ren C, Jin J, Li C, Xiang J, Wu Y, Zhou Y, et al. Metformin inactivates the cGAS-STING pathway through autophagy and suppresses senescence in nucleus pulposus cells. J Cell Sci. 2022;135:jcs259738. 10.1242/jcs.259738 35722742

[202] Carling GK, Fan L, Foxe NR, Norman K, Ye P, Wong MY, et al. Alzheimer’s disease-linked risk alleles elevate microglial cGAS-associated senescence and neurodegeneration in a tauopathy model. bioRxiv. 2024;2024.01.24.577107. 10.1016/j.neuron.2024.09.006 PMC11624100 39353433

[203] Gulen MF, Samson N, Keller A, Schwabenland M, Liu C, Glück S, et al. cGAS-STING drives ageing-related inflammation and neurodegeneration. Nature. 2023;620:374–80. 37532932 10.1038/s41586-023-06373-1 PMC10412454

[204] Liu R-M. Aging, Cellular Senescence, and Alzheimer’s Disease. Int J Mol Sci. 2022;23:1989. 35216123 10.3390/ijms23041989 PMC8874507

[205] Matsudaira T, Nakano S, Konishi Y, Kawamoto S, Uemura K, Kondo T, et al. Cellular senescence in white matter microglia is induced during ageing in mice and exacerbates the neuroinflammatory phenotype. Commun Biol. 2023;6:665. 37353538 10.1038/s42003-023-05027-2 PMC10290132

[206] Chinta SJ, Woods G, Rane A, Demaria M, Campisi J, Andersen JK. Cellular senescence and the aging brain. Exp Gerontol. 2015;68:3–7. 25281806 10.1016/j.exger.2014.09.018 PMC4382436

[207] Dou Z, Ghosh K, Vizioli MG, Zhu J, Sen P, Wangensteen KJ, et al. Cytoplasmic chromatin triggers inflammation in senescence and cancer. Nature. 2017;550:402–6. 28976970 10.1038/nature24050 PMC5850938

[208] Carreno G, Guiho R, Martinez-Barbera JP. Cell senescence in neuropathology: A focus on neurodegeneration and tumours. Neuropathol Appl Neurobiol. 2021;47:359–78. 33378554 10.1111/nan.12689 PMC8603933

[209] Si Z, Sun L, Wang X. Evidence and perspectives of cell senescence in neurodegenerative diseases. Biomed Pharmacother. 2021;137: 111327. 33545662 10.1016/j.biopha.2021.111327

[210] Ma X, Xin D, She R, Liu D, Ge J, Mei Z. Novel insight into cGAS-STING pathway in ischemic stroke: from pre- to post-disease. Front Immunol. 2023;14:1275408. 37915571 10.3389/fimmu.2023.1275408 PMC10616885

[211] Tchkonia T, Zhu Y, van Deursen J, Campisi J, Kirkland JL. Cellular senescence and the senescent secretory phenotype: therapeutic opportunities. J Clin Invest. 2013;123:966–72. 23454759 10.1172/JCI64098 PMC3582125

[212] De Cecco M, Ito T, Petrashen AP, Elias AE, Skvir NJ, Criscione SW, et al. L1 drives IFN in senescent cells and promotes age-associated inflammation. Nature. 2019;566:73–8. 30728521 10.1038/s41586-018-0784-9 PMC6519963

[213] Aguado J, Chaggar HK, Gómez-Inclán C, Shaker MR, Leeson HC, Mackay-Sim A, et al. Inhibition of the cGAS-STING pathway ameliorates the premature senescence hallmarks of Ataxia-Telangiectasia brain organoids. Aging Cell. 2021;20: e13468. 34459078 10.1111/acel.13468 PMC8441292

[214] Shippy DC, Ulland TK. Microglial Immunometabolism in Alzheimer’s Disease. Front Cell Neurosci. 2020;14: 563446. 33192310 10.3389/fncel.2020.563446 PMC7531234

[215] Heneka MT. Inflammasome activation and innate immunity in Alzheimer’s disease. Brain Pathol. 2017;27:220–2. 28019679 10.1111/bpa.12483 PMC8029274

[216] d’Errico P, Ziegler-Waldkirch S, Aires V, Hoffmann P, Mezö C, Erny D, et al. Microglia contribute to the propagation of Aβ into unaffected brain tissue. Nat Neurosci. 2022;25:20–5. 34811521 10.1038/s41593-021-00951-0 PMC8737330

[217] Xie L, Zhang N, Zhang Q, Li C, Sandhu AF, Iii GW, et al. Inflammatory factors and amyloid β-induced microglial polarization promote inflammatory crosstalk with astrocytes. Aging (Albany NY). 2020;12:22538–49. 33196457 10.18632/aging.103663 PMC7746366

[218] Huang Y, Liu B, Sinha SC, Amin S, Gan L. Mechanism and therapeutic potential of targeting cGAS-STING signaling in neurological disorders. Mol Neurodegener. 2023;18:79. 37941028 10.1186/s13024-023-00672-x PMC10634099

[219] Sala Frigerio C, Wolfs L, Fattorelli N, Thrupp N, Voytyuk I, Schmidt I, et al. The Major Risk Factors for Alzheimer’s Disease: Age, Sex, and Genes Modulate the Microglia Response to Aβ Plaques. Cell Rep. 2019;27:1293-1306.e6. 31018141 10.1016/j.celrep.2019.03.099 PMC7340153

[220] Escoubas CC, Dorman LC, Nguyen PT, Lagares-Linares C, Nakajo H, Anderson SR, et al. Type-I-interferon-responsive microglia shape cortical development and behavior. Cell. 2024;187:1936-1954.e24. 38490196 10.1016/j.cell.2024.02.020 PMC11015974

[221] Deczkowska A, Keren-Shaul H, Weiner A, Colonna M, Schwartz M, Amit I. Disease-Associated Microglia: A Universal Immune Sensor of Neurodegeneration. Cell. 2018;173:1073–81. 29775591 10.1016/j.cell.2018.05.003

[222] Yoh SM, Schneider M, Seifried J, Soonthornvacharin S, Akleh RE, Olivieri KC, et al. PQBP1 is a Proximal Sensor of the cGAS-dependent Innate Response to HIV-1. Cell. 2015;161:1293–305. 26046437 10.1016/j.cell.2015.04.050 PMC4503237

[223] Jin M, Shiwaku H, Tanaka H, Obita T, Ohuchi S, Yoshioka Y, et al. Tau activates microglia via the PQBP1-cGAS-STING pathway to promote brain inflammation. Nat Commun. 2021;12:6565. 34782623 10.1038/s41467-021-26851-2 PMC8592984

[224] Yeh FL, Hansen DV, Sheng M. TREM2, Microglia, and Neurodegenerative Diseases. Trends Mol Med. 2017;23:512–33. 28442216 10.1016/j.molmed.2017.03.008

[225] Villanelo F, Escalona Y, Pareja-Barrueto C, Garate JA, Skerrett IM, Perez-Acle T. Accessing gap-junction channel structure-function relationships through molecular modeling and simulations. BMC Cell Biol. 2017;18:5. 28124624 10.1186/s12860-016-0121-9 PMC5267332

[226] Ablasser A, Schmid-Burgk JL, Hemmerling I, Horvath GL, Schmidt T, Latz E, et al. Cell intrinsic immunity spreads to bystander cells via the intercellular transfer of cGAMP. Nature. 2013;503:530–4. 24077100 10.1038/nature12640 PMC4142317

[227] Zhou C, Chen X, Planells-Cases R, Chu J, Wang L, Cao L, et al. Transfer of cGAMP into Bystander Cells via LRRC8 Volume-Regulated Anion Channels Augments STING-Mediated Interferon Responses and Anti-viral Immunity. Immunity. 2020;52:767-781.e6. 32277911 10.1016/j.immuni.2020.03.016

[228] Elzinga SE, Henn R, Murdock BJ, Kim B, Hayes JM, Mendelson F, et al. cGAS/STING and innate brain inflammation following acute high-fat feeding. Front Immunol. 2022;13:1012594. 36248795 10.3389/fimmu.2022.1012594 PMC9556783

[229] Chang H, Li Z, Zhang W, Lin C, Shen Y, Zhang G, et al. Transfer of cGAMP from neuron to microglia activates microglial type I interferon responses after subarachnoid hemorrhage. Cell Commun Signal. 2024;22:3. 38169382 10.1186/s12964-023-01362-3 PMC10763285

[230] Koulakoff A, Mei X, Orellana JA, Sáez JC, Giaume C. Glial connexin expression and function in the context of Alzheimer’s disease. Biochim Biophys Acta. 2012;1818:2048–57. 22008509 10.1016/j.bbamem.2011.10.001

[231] Koulakoff A, Ezan P, Giaume C. Neurons control the expression of connexin 30 and connexin 43 in mouse cortical astrocytes. Glia. 2008;56:1299–311. 18512249 10.1002/glia.20698

[232] Mei X, Ezan P, Giaume C, Koulakoff A. Astroglial connexin immunoreactivity is specifically altered at β-amyloid plaques in β-amyloid precursor protein/presenilin1 mice. Neuroscience. 2010;171:92–105. 20813165 10.1016/j.neuroscience.2010.08.001

[233] Kalani K, Chaturvedi P, Chaturvedi P, Kumar Verma V, Lal N, Awasthi SK, et al. Mitochondrial mechanisms in Alzheimer’s disease: Quest for therapeutics. Drug Discov Today. 2023;28: 103547. 36871845 10.1016/j.drudis.2023.103547

[234] Haag SM, Gulen MF, Reymond L, Gibelin A, Abrami L, Decout A, et al. Targeting STING with covalent small-molecule inhibitors. Nature. 2018;559:269–73. 29973723 10.1038/s41586-018-0287-8

[235] Steinhagen F, Zillinger T, Peukert K, Fox M, Thudium M, Barchet W, et al. Suppressive oligodeoxynucleotides containing TTAGGG motifs inhibit cGAS activation in human monocytes. Eur J Immunol. 2018;48:605–11. 29215161 10.1002/eji.201747338 PMC6386451

[236] Li Q, Cao Y, Dang C, Han B, Han R, Ma H, et al. Inhibition of double-strand DNA-sensing cGAS ameliorates brain injury after ischemic stroke. EMBO Mol Med. 2020;12: e11002. 32239625 10.15252/emmm.201911002 PMC7136961

[237] Wang M, Sooreshjani MA, Mikek C, Opoku-Temeng C, Sintim HO. Suramin potently inhibits cGAMP synthase, cGAS, in THP1 cells to modulate IFN-β levels. Future Med Chem. 2018;10:1301–17. 29558821 10.4155/fmc-2017-0322

[238] An J, Woodward JJ, Sasaki T, Minie M, Elkon KB. Cutting edge: Antimalarial drugs inhibit IFN-β production through blockade of cyclic GMP-AMP synthase-DNA interaction. J Immunol. 2015;194:4089–93. 25821216 10.4049/jimmunol.1402793

[239] An J, Woodward JJ, Lai W, Minie M, Sun X, Tanaka L, et al. Inhibition of Cyclic GMP-AMP Synthase Using a Novel Antimalarial Drug Derivative in Trex1-Deficient Mice. Arthritis Rheumatol. 2018;70:1807–19. 29781188 10.1002/art.40559

[240] Wang X, Wang Y, Cao A, Luo Q, Chen D, Zhao W, et al. Development of cyclopeptide inhibitors of cGAS targeting protein-DNA interaction and phase separation. Nat Commun. 2023;14:6132. 37783727 10.1038/s41467-023-41892-5 PMC10545747

[241] Padilla-Salinas R, Sun L, Anderson R, Yang X, Zhang S, Chen ZJ, et al. Discovery of Small-Molecule Cyclic GMP-AMP Synthase Inhibitors. J Org Chem. 2020;85:1579–600. 31829590 10.1021/acs.joc.9b02666

[242] Green JP, El-Sharkawy LY, Roth S, Zhu J, Cao J, Leach AG, et al. Discovery of an inhibitor of DNA-driven inflammation that preferentially targets the AIM2 inflammasome. iScience. 2023;26:106758. 10.1016/j.isci.2023.106758 PMC10193008 37216118

[243] Vincent J, Adura C, Gao P, Luz A, Lama L, Asano Y, et al. Small molecule inhibition of cGAS reduces interferon expression in primary macrophages from autoimmune mice. Nat Commun. 2017;8:750. 28963528 10.1038/s41467-017-00833-9 PMC5622107

[244] Hall J, Brault A, Vincent F, Weng S, Wang H, Dumlao D, et al. Discovery of PF-06928215 as a high affinity inhibitor of cGAS enabled by a novel fluorescence polarization assay. PLoS ONE. 2017;12: e0184843. 28934246 10.1371/journal.pone.0184843 PMC5608272

[245] Lama L, Adura C, Xie W, Tomita D, Kamei T, Kuryavyi V, et al. Development of human cGAS-specific small-molecule inhibitors for repression of dsDNA-triggered interferon expression. Nat Commun. 2019;10:2261. 31113940 10.1038/s41467-019-08620-4 PMC6529454

[246] Zhao W, Xiong M, Yuan X, Li M, Sun H, Xu Y. In Silico Screening-Based Discovery of Novel Inhibitors of Human Cyclic GMP-AMP Synthase: A Cross-Validation Study of Molecular Docking and Experimental Testing. J Chem Inf Model. 2020;60:3265–76. 32459092 10.1021/acs.jcim.0c00171

[247] Tan J, Wu B, Chen T, Fan C, Zhao J, Xiong C, et al. Synthesis and Pharmacological Evaluation of Tetrahydro-γ-carboline Derivatives as Potent Anti-inflammatory Agents Targeting Cyclic GMP-AMP Synthase. J Med Chem. 2021;64:7667–90. 34044539 10.1021/acs.jmedchem.1c00398

[248] Li J, Xiong M, Liu J, Zhang F, Li M, Zhao W, et al. Discovery of novel cGAS inhibitors based on natural flavonoids. Bioorg Chem. 2023;140: 106802. 37666112 10.1016/j.bioorg.2023.106802

[249] Liu Z-S, Cai H, Xue W, Wang M, Xia T, Li W-J, et al. G3BP1 promotes DNA binding and activation of cGAS. Nat Immunol. 2019;20:18–28. 30510222 10.1038/s41590-018-0262-4 PMC8276115

[250] Yu Z, Zheng L, Geng Y, Zhang Y, Wang Y, You G, et al. FTO alleviates cerebral ischemia/reperfusion-induced neuroinflammation by decreasing cGAS mRNA stability in an m6A-dependent manner. Cell Signal. 2023;109: 110751. 37321527 10.1016/j.cellsig.2023.110751

[251] Sun H, Huang Y, Mei S, Xu F, Liu X, Zhao F, et al. A Nuclear Export Signal Is Required for cGAS to Sense Cytosolic DNA. Cell Rep. 2021;34: 108586. 33406424 10.1016/j.celrep.2020.108586

[252] Liu M, Li Y, Han S, Wang H, Li J. Activin A alleviates neuronal injury through inhibiting cGAS-STING-mediated autophagy in mice with ischemic stroke. J Cereb Blood Flow Metab. 2023;43:736–48. 36537048 10.1177/0271678X221147056 PMC10108189

[253] Chauhan C, Kaundal RK. The role of cGAS-STING signaling in ischemic stroke: From immune response to therapeutic targeting. Drug Discov Today. 2023;28: 103792. 37783431 10.1016/j.drudis.2023.103792

[254] Li S, Hong Z, Wang Z, Li F, Mei J, Huang L, et al. The Cyclopeptide Astin C Specifically Inhibits the Innate Immune CDN Sensor STING. Cell Rep. 2018;25:3405-3421.e7. 30566866 10.1016/j.celrep.2018.11.097

[255] Siu T, Altman MD, Baltus GA, Childers M, Ellis JM, Gunaydin H, et al. Discovery of a Novel cGAMP Competitive Ligand of the Inactive Form of STING. ACS Med Chem Lett. 2019;10:92–7. 30655953 10.1021/acsmedchemlett.8b00466 PMC6331172

[256] Hong Z, Mei J, Li C, Bai G, Maimaiti M, Hu H, et al. STING inhibitors target the cyclic dinucleotide binding pocket. Proc Natl Acad Sci U S A. 2021;118: e2105465118. 34099558 10.1073/pnas.2105465118 PMC8214703

[257] Hansen AL, Buchan GJ, Rühl M, Mukai K, Salvatore SR, Ogawa E, et al. Nitro-fatty acids are formed in response to virus infection and are potent inhibitors of STING palmitoylation and signaling. Proc Natl Acad Sci U S A. 2018;115:E7768–75. 30061387 10.1073/pnas.1806239115 PMC6099880

[258] Mukai K, Konno H, Akiba T, Uemura T, Waguri S, Kobayashi T, et al. Activation of STING requires palmitoylation at the Golgi. Nat Commun. 2016;7:11932. 27324217 10.1038/ncomms11932 PMC4919521

[259] Vinogradova EV, Zhang X, Remillard D, Lazar DC, Suciu RM, Wang Y, et al. An Activity-Guided Map of Electrophile-Cysteine Interactions in Primary Human T Cells. Cell. 2020;182:1009-1026.e29. 32730809 10.1016/j.cell.2020.07.001 PMC7775622

[260] Kwon D, Park E, Sesaki H, Kang S-J. Carbonyl cyanide 3-chlorophenylhydrazone (CCCP) suppresses STING-mediated DNA sensing pathway through inducing mitochondrial fission. Biochem Biophys Res Commun. 2017;493:737–43. 28859978 10.1016/j.bbrc.2017.08.121

[261] Li W, Li Y, Kang J, Jiang H, Gong W, Chen L, et al. 4-octyl itaconate as a metabolite derivative inhibits inflammation via alkylation of STING. Cell Rep. 2023;42: 112145. 36862550 10.1016/j.celrep.2023.112145

[262] Prabakaran T, Troldborg A, Kumpunya S, Alee I, Marinković E, Windross SJ, et al. A STING antagonist modulating the interaction with STIM1 blocks ER-to-Golgi trafficking and inhibits lupus pathology. EBioMedicine. 2021;66: 103314. 33813142 10.1016/j.ebiom.2021.103314 PMC8047499

[263] Jia M, Qin D, Zhao C, Chai L, Yu Z, Wang W, et al. Redox homeostasis maintained by GPX4 facilitates STING activation. Nat Immunol. 2020;21:727–35. 32541831 10.1038/s41590-020-0699-0

[264] Gao J, Zheng M, Wu X, Zhang H, Su H, Dang Y, et al. CDK inhibitor Palbociclib targets STING to alleviate autoinflammation. EMBO Rep. 2022;23: e53932. 35403787 10.15252/embr.202153932 PMC9171422

[265] Liu J, Yuan L, Ruan Y, Deng B, Yang Z, Ren Y, et al. Novel CRBN-Recruiting Proteolysis-Targeting Chimeras as Degraders of Stimulator of Interferon Genes with In Vivo Anti-Inflammatory Efficacy. J Med Chem. 2022;65:6593–611. 35452223 10.1021/acs.jmedchem.1c01948

[266] Liu H, Wang F, Cao Y, Dang Y, Ge B. The multifaceted functions of cGAS. J Mol Cell Biol. 2022;14:mjac031. 10.1093/jmcb/mjac031 PMC9475664 35536585

[267] Li Q, Wu P, Du Q, Hanif U, Hu H, Li K. cGAS-STING, an important signaling pathway in diseases and their therapy. MedComm. 2020;2024(5): e511. 10.1002/mco2.511 PMC10960729 38525112

[268] Fryer AL, Abdullah A, Taylor JM, Crack PJ. The Complexity of the cGAS-STING Pathway in CNS Pathologies. Front Neurosci. 2021;15: 621501. 33633536 10.3389/fnins.2021.621501 PMC7900568

[269] Wang R, Hussain A, Guo Q, Ma M. cGAS-STING at the crossroads in cancer therapy. Crit Rev Oncol Hematol. 2024;193: 104194. 37931770 10.1016/j.critrevonc.2023.104194

[270] Joshi B, Joshi JC, Mehta D. Regulation of cGAS Activity and Downstream Signaling. Cells. 2022;11:2812. 36139387 10.3390/cells11182812 PMC9496985

[271] Zhang Z, Yuan B, Bao M, Lu N, Kim T, Liu Y-J. The helicase DDX41 senses intracellular DNA mediated by the adaptor STING in dendritic cells. Nat Immunol. 2011;12:959–65. 21892174 10.1038/ni.2091 PMC3671854

[272] Singh RS, Vidhyasagar V, Yang S, Arna AB, Yadav M, Aggarwal A, et al. DDX41 is required for cGAS-STING activation against DNA virus infection. Cell Rep. 2022;39: 110856. 35613581 10.1016/j.celrep.2022.110856 PMC9205463

[273] Zhu Z, Lu H, Jin L, Gao Y, Qian Z, Lu P, et al. C-176 loaded Ce DNase nanoparticles synergistically inhibit the cGAS-STING pathway for ischemic stroke treatment. Bioact Mater. 2023;29:230–40. 37502677 10.1016/j.bioactmat.2023.07.002 PMC10371767

[274] Nance E, Pun SH, Saigal R, Sellers DL. Drug delivery to the central nervous system. Nat Rev Mater. 2022;7:314–31. 38464996 10.1038/s41578-021-00394-w PMC10923597

[275] Terstappen GC, Meyer AH, Bell RD, Zhang W. Strategies for delivering therapeutics across the blood-brain barrier. Nat Rev Drug Discov. 2021;20:362–83. 33649582 10.1038/s41573-021-00139-y

[276] Cai H, Pang Y, Ren Z, Fu X, Jia L. Delivering synaptic protein mRNAs via extracellular vesicles ameliorates cognitive impairment in a mouse model of Alzheimer’s disease. BMC Med. 2024;22:138. 38528511 10.1186/s12916-024-03359-2 PMC10964680

